# HIDANet: a lightweight deep learning framework for Vannamei post-larval stage classification and morphometric estimation with background bias validation

**DOI:** 10.3389/frai.2026.1821929

**Published:** 2026-07-23

**Authors:** Sugunapriya A, Markkandan S

**Affiliations:** School of Electronics Engineering, Vellore Institute of Technology, Chennai, Tamil Nadu, India

**Keywords:** aquaculture automation, attention mechanism, convolutional neural network, hatchery monitoring, *Litopenaeus vannamei*, morphometric analysis, post-larval staging

## Abstract

**Introduction:**

Quality control of hatchery production relies on accurate developmental staging of the Pacific white shrimp *Litopenaeus vannamei* post-larvae (PL), but current methods rely on subjective manual visual evaluation that leads to observer bias and inconsistency.

**Methods:**

In this study, the Hierarchical Isotropic Dense Attention Network (HIDANet) has been introduced, a lightweight convolutional neural network with 0.033M parameters that learns to classify seven post-larval stages (PL5–PL12) in a digitally obtained image of a defined larva. Its architecture uses multibranch isotropic depthwise separable convolution, channel-wise concatenation, and a Convolutional Block Attention Module (CBAM) to recalibrate sequential channel and spatial features. The background color bias was removed using an aggressive augmentation scheme that included strong color jittering, stochastic grayscale conversion, random auto-contrast, and geometric perturbations with inverse-frequency weighted sampling to address class imbalance.

**Results:**

HIDANet trained on 5,835 collected hatchery images reached a test accuracy of 98.44% with color inputs and 96.89% with grayscale inputs, with a macro-averaged F1-score of 0.99, which confirms strong morphological learning that is independent of chromatic background signals. An end-to-end workflow combining CLAHE image enhancement, Gaussian adaptive thresholding, and skeleton-based morphometric filtering offers automated larva counting, area and length measurements of the population, and density categorized into three levels (Low, Medium, and High).

**Discussion:**

The suggested framework allows for classifying the stages of PL and assessing larval numbers in the same image simultaneously, which is a scalable and low-cost method of quality monitoring in commercial aquaculture systems in real time.

## 1 Introduction

Shrimp farming has become one of the fastest-growing industries in world food production, with an annual production of more than 5 million metric tons, contributing to food security and economic growth of coastal communities worldwide (FAO, 2023, 2024). The dominant species used for culture is *Penaeus vannamei*, which is known for its rapid growth rate, disease resistance, and tolerance to different culture systems (Neiland et al., 2001). However, some of the challenges that persist and limit the efficiency and profitability of culture production include larval quality, stocking efficiency, and disease management (Moss et al., 2012; Flegel, 2019; N'Souvi et al., 2024).

Post-larval (PL) developmental staging refers to the identification of shrimp developmental stages after the larval phase based on morphological features. In Pacific white shrimp, *Litopenaeus vannamei*, these stages are denoted as PL1 through PL12 and are significant developmental milestones prior to being stocked in grow-out ponds. Accurate identification of different developmental stages, ranging from PL5 to PL12, is crucial to determine the optimum stocking time, the quality of the larvae, and the implementation of appropriate feeding and biosecurity measures (FAO, 2003; Racotta et al., 2004). Conventional identification of developmental stages is based on manual microscopic analysis by skilled personnel to identify morphological features such as body length, presence of the rostrum, and color patterns of the chromatophores. This approach is subjective, labor-intensive, and prone to inter-observer variability, particularly when differentiating visually similar developmental stages (Saberioon et al., 2017).

Artificial intelligence and computer vision have opened new avenues for automation and precision management in aquaculture (Rather et al., 2024; Li and Du, 2022; Aung et al., 2025). Recent advances in deep learning have demonstrated strong performance in diverse aquaculture tasks, but existing studies concentrate on counting or detection tasks in isolation, with limited attention paid to the critical issue of fine-grained developmental stage recognition across the complete post-larval spectrum (PL5–PL12) (Saberioon et al., 2017).

One of the most fundamental issues in applying computer vision to the classification of biological images is the possibility of learning background biases or spurious correlations instead of true morphological characteristics, such as correlations with background color or other imaging artifacts (Geirhos et al., 2020, 2018). Such background biases can significantly hamper the generalization of models to real-world hatchery environments, which can differ from farm to farm and from stage to stage (Beery et al., 2018). Despite its significance, explicit validation of background bias remains largely absent in the aquaculture computer vision literature, undermining the reliability of automated systems in production settings. Furthermore, practical deployment in resource-constrained hatchery environments demands lightweight architectures that balance classification performance with computational efficiency (Sandler et al., 2018; Han et al., 2020; Chahbouni et al., 2025).

Lightweight convolutional neural network (CNN) architectures have demonstrated the potential to achieve this balance, yet their application to fine-grained shrimp larvae stage classification with explicit background bias validation remains unexplored. This study bridges these gaps by offering the following contributions:

1. Accurate multiclass post-larval stage classification using a hybrid lightweight CNN model that combines depthwise separable convolutions, dense connectivity, and attention mechanisms.
2. Skeleton-based anchor-free larvae density estimation for population analysis.
3. Rigorous background bias verification with grayscale analysis and Grad-CAM visualization.
4. Detailed model component ablation analysis to identify the contribution of each architecture layer.
5. Comprehensive comparative evaluation against eight state-of-the-art CNN models on a range of robustness metrics and computational efficiency analysis.

The key contribution indicates that morphological features can be learned well without relying on the differences in background colors, with high classification accuracy in a computationally efficient model suitable for deployment in resource-constrained real-world hatchery environments.

The remainder of this paper is organized as follows. Section 2 provides an overview of earlier research in the context of shrimp post-larval (PL) stage classification and detection by leveraging computer vision and deep learning technologies. Section 3 presents the hatchery image data utilized in the current research, such as the data collection and distribution of classes in stages PL5–PL12. Section 4 introduces the suggested methodology, which includes the HIDANet architecture, background bias validation, and morphometric analysis of the predicted PL stage. Section 5 outlines the experimental setup and evaluation measures to assess model performance. Section 6 reports the classification results and performance comparisons. Section 7 addresses the effectiveness of the suggested framework and its implications in the context of hatchery monitoring, and Section 8 summarizes the study and notes possible future research directions.

## 2 Related work

### 2.1 Image-based shrimp monitoring

In recent years, there has been a growing interest in automated image analysis for shrimp aquaculture. Saleh et al. (2024) introduced a CNN-based regression model and demonstrated the feasibility of non-contact prawn size and weight estimation. Correia et al. (2025) conducted a systematic literature review on image-based shrimp surveillance methods and mapped the research landscape in shrimp farming. Zhou et al. (2024) worked on density-mapped regression for larvae counting by avoiding direct bounding box detection. Duan et al. (2024) improved YOLOv5 with dynamic anchor adjustment for shrimp larvae detection, and Peng et al. (2024) presented multiscale attention fusion networks with better robustness under low-contrast water conditions. Certain works contributed to larval localization with domain-specific detection frameworks, including Tiyapunjanit et al. (2023), CAGNet (Zhang et al., 2024), and multiscale feature fusion networks (Dang et al., 2025).

Concurrent research has concentrated on biomass and weight estimation, which are essential metrics for aquaculture management. Chirdchoo et al. (2024) developed a deep learning model integrating Detectron2 and an artificial neural network (ANN) for morphometric feature extraction, achieving R^2^ of 94.50% for live Pacific white shrimp weight estimation in clay pond environments. Ramírez-Coronel et al. (2024) provided labeled images for biomass estimation and contributed a dataset with 5507 images of *Litopenaeus vannamei*. In a recent study, Zhang S. et al. (2025) inferred the use of multimodal strategies that combined video and sensor data for shrimp biomass estimation. To enable the comparison of classification and detection models, a special benchmark dataset Lanke et al. (2023) of 10,000 samples of Pacific White Shrimp, Tiger Prawn, and Ghost Shrimp at various stages of life was created. A lightweight multiscale detection framework used in quality control applications, namely, automated shrimp meat inspection in food processing environments, was suggested based on YOLOv11 (Zhang H. et al., 2025), and automated morphometric measurement in grow-out settings was performed using a real-time underwater monitoring framework that combined YOLOv5-OBB localization with YOLOv8-Seg abdominal segmentation (Vadde et al., 2025).

Although this is a rich range of activity covering counting, biomass estimation, species classification, and quality inspection, none of the studies cover post-larval developmental stage classification, which necessitates that morphological distinction across visually similar intra-species stages be finely discriminated under variable hatchery imaging conditions.

### 2.2 Learning through spurious correlations and background bias

One of the most documented problems with deep CNN implementation is shortcut learning, when the model uses spurious correlations found in training data instead of features relevant to the task (Geirhos et al., 2020). Beery et al. (2018) showed that models trained on controlled imaging settings cannot generalize to different background statistics, which is directly applicable to the hatchery imaging situation where the turbidity of water, type of container, and lighting conditions vary across farms and stages of development. Geirhos et al. (2018) also demonstrated that ImageNet-pretrained CNNs are highly biased in texture and color, indicating that background dependency can be transferred via transfer learning from natural image datasets to domain-specific tasks. These results encourage the explicit bias assessment as a component of model validation that is performed in this study using grayscale ablation analysis.

### 2.3 Lightweight CNN architectures

The design of computationally efficient CNN architectures has been an active research direction driven by edge deployment requirements (Howard et al., 2017). MobileNetV2 (Sandler et al., 2018) introduced inverted residuals and linear bottlenecks to reduce the computational cost while maintaining representational capacity. MobileNetV3 (Howard et al., 2019) further incorporated neural architecture search and hard-swish activations to improve efficiency. ShuffleNetV2 (Ma et al., 2018) proposed channel splitting and shuffling operations as practical guidelines for lightweight designs. SqueezeNet (Iandola et al., 2016) demonstrated AlexNet-level accuracy with 50 × fewer parameters through fire-module compression. Deeper networks such as ResNet (He et al., 2016) and DenseNet (Huang et al., 2017) improve feature representation using residual and dense connections; however, despite their representational capacity, these models may not be sufficiently accurate for distinguishing the morphological details necessary for shrimp post-larval developmental stage classification. Liu et al. (2022) modernized the standard CNN design by incorporating transformer-inspired architectural elements. These architectures served as comparative baselines in this study.

From the literature review (Table 1), it is clear that although great progress has been made in shrimp larvae analysis, some critical limitations still need to be addressed. The majority of the existing literature only classifies shrimp larvae into a few broad categories and does not address the entire range of post-larval stages. Moreover, the tasks of counting and stage classification are usually considered as two separate problems, leading to systems that do not have an integrated framework for recognizing stages and estimating larval density simultaneously. The effects of background bias and shortcut learning are also not considered in the majority of existing literature on aquaculture vision applications. In addition, existing literature is based on laboratory datasets, and there is a lack of validation in real-world hatchery settings with varying lighting, turbidity, and imaging systems. The above issues are addressed in the current study by providing an integrated framework that involves fine-grained multiclass classification (PL5–PL12), skeleton-based larvae counting (Zhang and Suen, 1984), background bias validation using grayscale testing and Grad-CAM analysis (Selvaraju et al., 2017), and multimetric evaluation.

**Table 1:** Key studies in shrimp larvae analysis.

Study	Task	Method	Key contribution	Limitation
Zhou et al. (2024)	Counting	Density map regression	Accurate counting and sizing in dense larvae populations	No stage classification
Duan et al. (2024)	Detection/counting	Improved YOLOv5 with regional segmentation	Accurate counting of densely packed shrimp larvae	Stage information absent
Peng et al. (2024)	Counting	Multiscale attention fusion	Handles overlapping larvae populations	Counting only
Wang et al. (2024)	Counting	Semi-supervised learning	Reduced labeling effort	No stage recognition
Bukas et al. (2024)	Evaluation	DL benchmark	Real-world hatchery evaluation	Not stage-specific
Qian et al. (2023)	Counting	Optical vision + ML	Automated counting platform	Limited scalability
Tiyapunjanit et al. (2023)	Classification	Probabilistic DL	Susceptible larvae detection	Binary classification
Melinda et al. (2024)	Classification	CNN	Cultivation outcome classification	Coarse categories
Zhang et al. (2024)	Detection	Anchor-free Network	Improved larvae detection	No density estimation
Dang et al. (2025)	Detection	Multiscale fusion	Robust localization	No stage classification

## 3 Dataset

**Figure 1:**
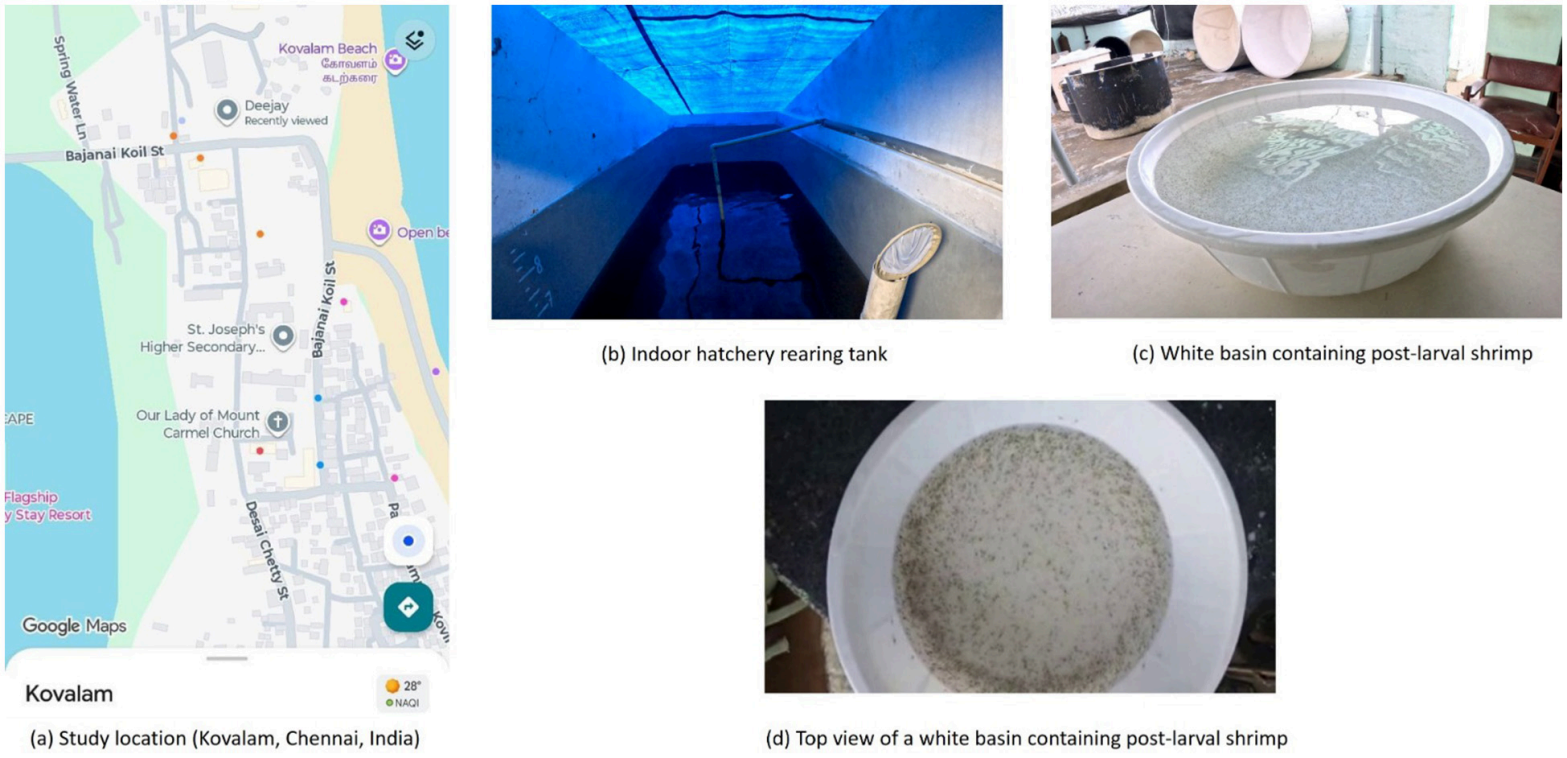
Controlled tank environment of Penaeidae post-larval growth in DeeJay Shrimp Hatchery, Chennai, Tamil Nadu, India. Maps data © 2026 Google.

The dataset used for the proposed study was obtained from the post-larval (PL) shrimp belonging to the Penaeidae family (*Penaeus vannamei*). Data collection was conducted on a commercial aquaculture farm in Deejay Hatchery, Kovalam, Chennai, India, where the hatchery operates under controlled conditions (Figure 1). The life cycle of shrimp consists of several major ontogenetic phases, with each stage represented by unique morphological and physiological characteristics (including egg, nauplius, zoea, mysis, and PL). PL stages were chosen in the proposed study, and they play a vital role in growth and stocking assessment, where each stage has its own morphological identity. Each stage is cultivated in specific tanks to provide appropriate environmental and nutritional conditions. The feeding pattern adhered to hatchery guidelines, incorporating microalgae and formulated diets to ensure balanced growth and reduce stress. The lighting conditions were controlled by the hatchery, and the background conditions were standardized during image acquisition to facilitate visual consistency and noise reduction.

**Figure 2:**
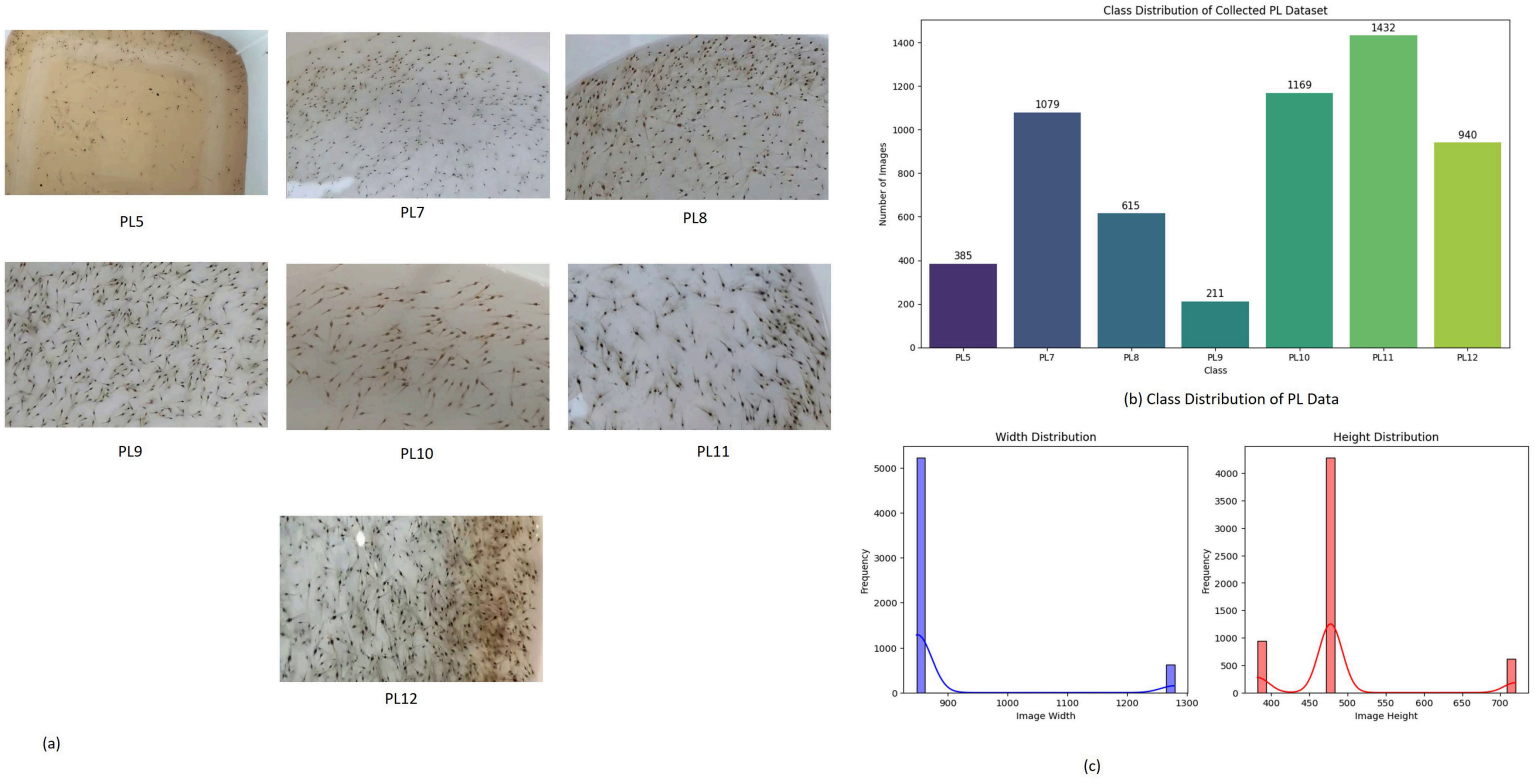
Dataset characteristics of the collected shrimp post-larval (PL) images. **(a)** Representative samples of the collected hatchery dataset showing different post-larval developmental stages of *Litopenaeus vannamei*. **(b)** Class distribution of PL data. **(c)** Width and height distribution of PL data.

However, natural biological and process variations were introduced as inherent class imbalances in PL stages. Specifically, the variations in the background color shown in Figure 2 realistically represent the aquaculture settings. PL5 early stage larvae are nourished in enriched water at a higher phytoplankton concentration, producing a brownish or greenish background color. PL7–PL12 are elevated to clearer water later due to their feeding habits and increased larval mass, resulting in light backgrounds. These variations provide realistic data on which to test the models in a robust manner.

The dataset contained 5,835 images representing seven post-larval developmental stages (PL5 and PL7–PL12). Representative samples and dataset statistics are illustrated in Figure 2, and the detailed class distribution is summarized in Table 2. For model development and evaluation, stratified sampling was employed to partition the dataset into train, validation, and test sets in a split ratio of 70:20:10. This stratified division preserved the class proportions across all subsets. It should be noted that PL6 specimens were not available during the data collection period, and the implications of this are discussed in Section 7.2.

**Table 2:** Class distribution of collected PL dataset.

Class	PL5	PL7	PL8	PL9	PL10	PL11	PL12	Total
Images	385	1,079	615	214	1,170	1,432	940	**5,835**

Bold value indicates the total number of images in the dataset.

## 4 Materials and methods

### 4.1 Data augmentation strategy

To enhance model robustness and feature learning with reduced background bias, a staged data augmentation strategy Cubuk et al. (2019); Hendrycks et al. (2020); Shorten and Khoshgoftaar (2019) consisting of baseline, moderate, and strong augmentation was adopted. The baseline preprocessing included resizing images to 224 × 224 pixels and normalization using ImageNet statistics, which refers to standardizing the pixel intensity values of input images using the mean and standard deviation computed from the ImageNet dataset for each RGB channel. Specifically, each channel is normalized using mean values [0.485, 0.456, 0.406] and standard deviation values [0.229, 0.224, 0.225], ensuring consistent input scaling and improving training stability and convergence. To improve the robustness against natural variations in hatchery environments, moderate geometric transformations were introduced, including random rotations within ±20° and random-resized cropping with a scale sampled from *A* ~ U(0.49, 1.0), followed by bilinear resizing to the network input resolution.

To further prevent the model from exploiting background color differences across developmental stages, a strong color-invariant augmentation strategy was applied during training. Brightness, contrast, and saturation were randomly perturbed by up to ±50%, while the hue was shifted within ±30°. Additionally, 30% of the images were converted to grayscale, and random autocontrast was applied to 20% of the samples to diversify the illumination conditions. Brightness and contrast transformations are expressed in Equations 1 and 2 as


$$\begin{array}{l} I_{\text{out}}=I_{\text{in}}+\beta ,\;\beta \sim U\left(-0.5,0.5\right) \end{array}$$ (1)

$$\begin{array}{l} I_{\text{out}}=\gamma I_{\text{in}}+\left(1-\gamma \right)\mu ,\;\gamma \sim U\left(0.5,1.5\right) \end{array}$$ (2)

where *I*_*in*_ and *I*_*out*_ denote input and transformed intensities, and γ is a blending factor sampled from a uniform distribution U(0.5, 1.5), and μ is the mean image intensity. The combined augmentation pipeline improves invariance to illumination, orientation, and scale variations while encouraging the network to focus on biologically meaningful morphological characteristics rather than background artifacts.

### 4.2 Proposed methodology

**Figure 3:**
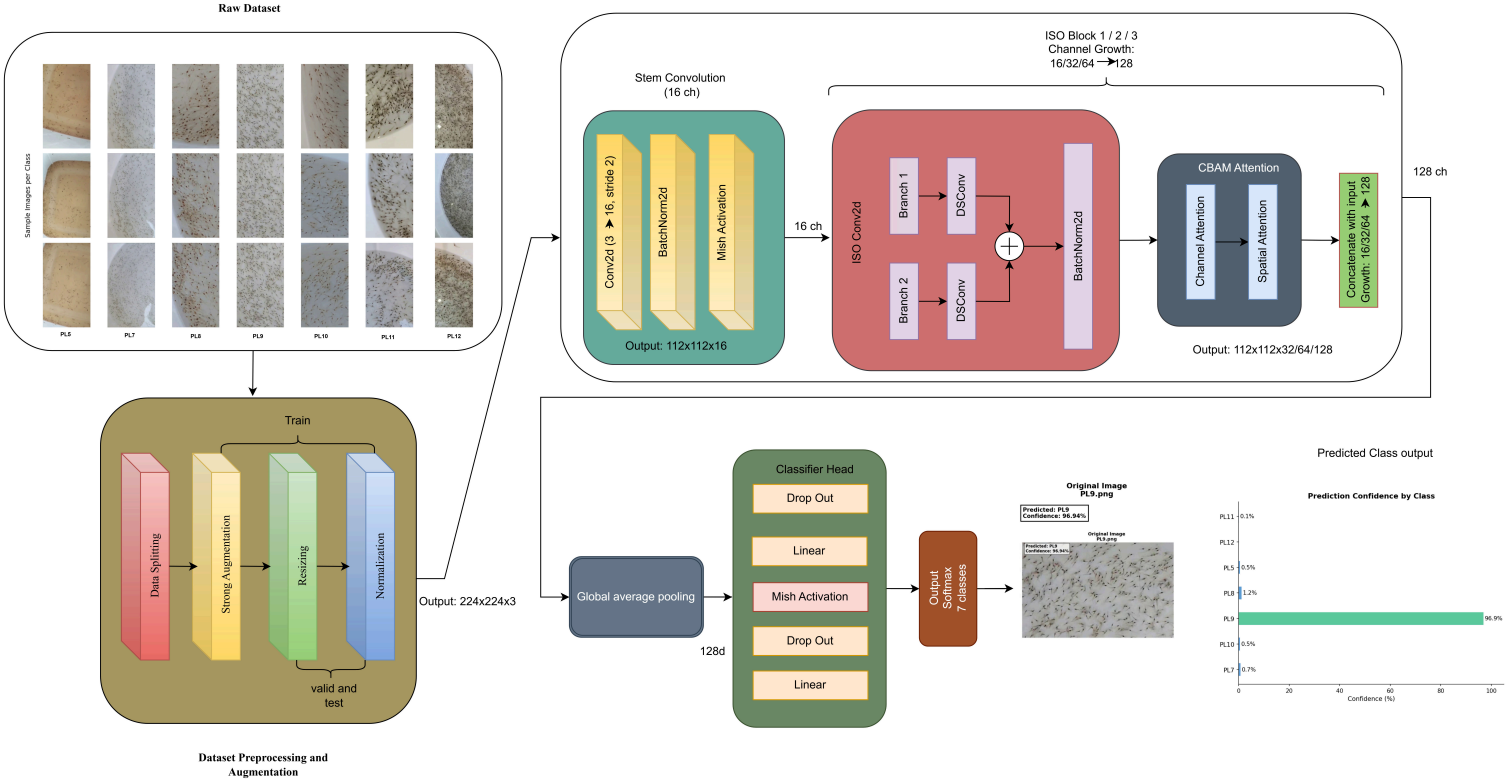
Architecture of the proposed HIDANet model for the classification of shrimp PL stages.

To achieve accurate and computationally efficient classification of underwater shrimp post-larval (PL) stages, we propose a Hierarchical Isotropic Depthwise Attention Network, HIDANet, as shown in Figure 3. Inspired by earlier baseline models, the HIDANet integrates multibranch depthwise-separable convolutions, localized dense feature reuse, and attention-based feature refinement (Woo et al., 2018; Chahbouni et al., 2025) within a compact framework containing 0.033M trainable parameters. This design is highly focused on underwater digital imagery, where illumination variability, turbidity, and subtle interstage morphological differences present significant challenges. The complete processing of HIDANet is defined in Equation 3 as


$$\begin{array}{r} I_{224\times 224\times 3}\to \text{Stem}\to {\text{ISOBlock}}_{1}\to {\text{ISOBlock}}_{2}\to {\text{ISOBlock}}_{3}\\ \to \text{GAP}\to \text{Classifier}\to ŷ\in {\mathbb{R}}^{7} \end{array}$$ (3)

where ŷ denotes the predicted logits of the seven PL classes.

#### 4.2.1 Stem, initial feature extraction module

The network begins with a stem module that performs initial feature extraction and spatial downsampling that extracts low-level visual patterns using a 3 × 3 convolution with a stride of 2, followed by Batch Normalization (BN) in Equation 4 and Mish activation in Equation 5:


$$\begin{array}{l} {\text{F}}_{0}=\text{Mish}\left(\text{BN}\left(W_{s}*I\right)\right) \end{array}$$ (4)

where $W_{s}\in {\mathbb{R}}^{16\times 3\times 3\times 3}$ denotes the learnable convolutional kernels, and * represents the convolution. The Mish activation is defined as


$$\begin{array}{l} \text{Mish}\left(x\right)=x\cdot \tanh\left(\ln\left(1+e^{x}\right)\right) \end{array}$$ (5)

This stage reduces the spatial resolution from 224 × 224 to 112 × 112 while generating 16 feature maps that capture low-level edge and texture information.

#### 4.2.2 Integrated spatial operation block (ISOBlock)

**Figure 4:**
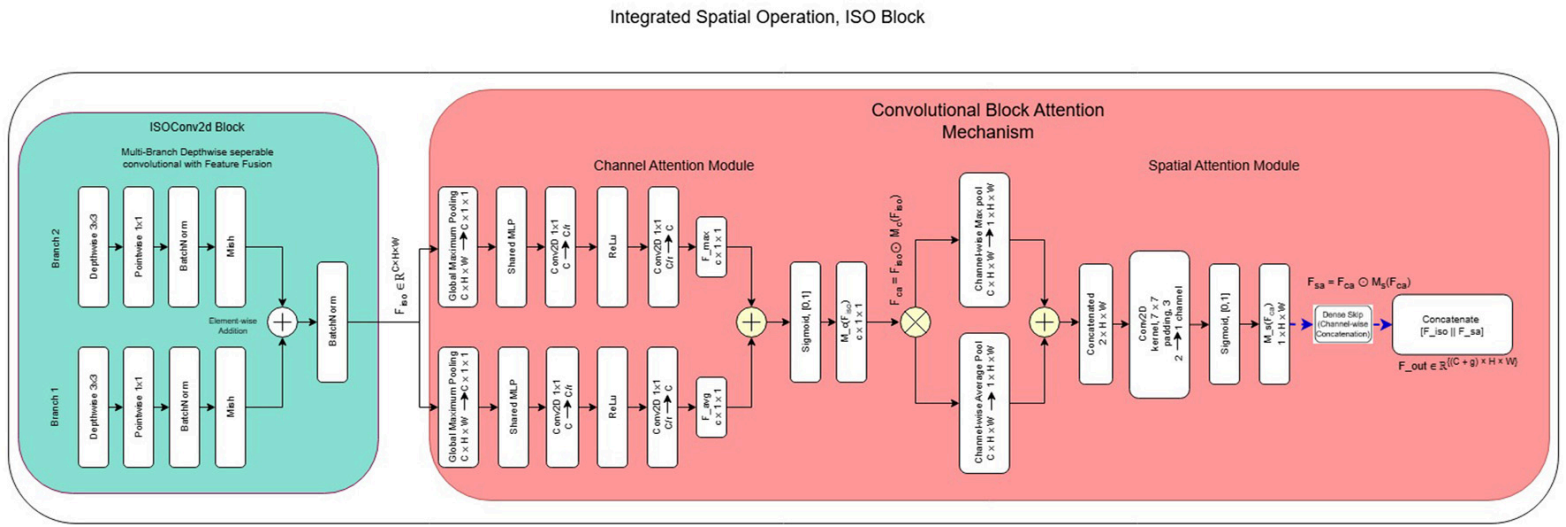
Proposed ISOBlock architecture with CBAM integration.

The core computational unit of HIDANet is the ISOBlock, as illustrated in Figure 4. It is designed to preserve spatial resolution while enriching feature representations. Unlike conventional architectures that aggressively downsample feature maps, ISOBlocks maintain spatial consistency to retain fine-grained morphological details that are essential for recognizing small or low-contrast larvae. Each ISOBlock consists of multibranch depthwise separable convolutional units (ISOConv2D) followed by a Convolutional Block Attention Module (CBAM). The multibranch design enables the extraction of diverse feature patterns at the same resolution, whereas depthwise separable convolutions reduce computational complexity. The CBAM further refines the feature maps by sequentially applying channel and spatial attention, allowing the network to focus on the most informative morphological regions. Across the three stacked ISOBlocks, feature channels expand geometrically as expressed in Equation 6


$$\begin{array}{l} 16\to 32\to 64\to 128 \end{array}$$ (6)

This progressive expansion of channels enables the network to learn hierarchical feature representations at different abstraction levels. In the initial layers, the network focuses on capturing low-level visual cues such as edges, textures, and simple structural patterns. As the network depth increases, higher-level layers learn more complex morphological characteristics associated with shrimp post-larval stages (PL5 and PL7–PL12). This gradual increase in feature dimensionality enhances the representational capacity of the model while maintaining computational efficiency through depthwise separable convolutions.

##### 4.2.2.1 Multibranch depthwise convolution

Within each ISOConv2D unit, two parallel depthwise separable branches independently extract spatial features, and their outputs are fused via element-wise addition before a shared BatchNorm layer. This intra-block additive fusion aggregates multiscale representations without expanding the channel dimension. For branch *b*, the output is defined as in Equation 7


$$\begin{array}{l} {\text{F}}_{b}=\text{Mish}\left(\text{BN}\left({\text{PW}}_{b}\left({\text{DW}}_{b}\left({\text{F}}_{\text{in}}\right)\right)\right)\right) \end{array}$$ (7)

where ${\text{F}}_{in}\in {\mathbb{R}}^{C\times H\times W}$, with *C*, *H*, and *W* denoting the number of input channels, height, and width, respectively, and *b* ∈ {1, …, *B*} where *B* is the total number of parallel branches.

The depthwise separable convolution is given by in Equation 8


$$\begin{array}{l} {\text{DW}}_{b}\left({\text{F}}_{\text{in}}\right)={\text{F}}_{\text{in}}*{\text{K}}_{b}^{\left(d\right)},\;{\text{K}}_{b}^{\left(d\right)}\in {\mathbb{R}}^{C\times 1\times k\times k} \end{array}$$ (8)

where * denotes the convolution operator and *k* is the spatial kernel size, which applies one *k*×*k* filter per input channel. This operation captures spatial information independently within each channel. The subsequent pointwise convolution performs channel mixing using Equation 9,


$$\begin{array}{l} {\text{PW}}_{b}\left(\text{X}\right)=\text{X}*{\text{K}}_{b}^{\left(p\right)},\;{\text{K}}_{b}^{\left(p\right)}\in {\mathbb{R}}^{C_{\text{out}}\times C\times 1\times 1} \end{array}$$ (9)

where *C*_out_ denotes the number of output channels. Decoupling spatial and channel mixing operations, computational complexity is reduced as expressed in Equation 10.


$$\begin{array}{l} O\left(CC^{\prime }k^{2}\right)\;\text{to }O\left(Ck^{2}+CC^{\prime }\right) \end{array}$$ (10)

where $C^{\prime }=C_{\text{out}}$ denotes the number of output channels. The outputs of all *B* branches is defined in Equation 11 as,


$$\begin{array}{l} {\text{F}}_{\text{iso}}=\phi \left({\text{F}}_{1},{\text{F}}_{2},\ldots ,{\text{F}}_{B}\right) \end{array}$$ (11)

where ${\text{F}}_{\text{iso}}\in {\mathbb{R}}^{C_{\text{out}}\times H\times W}$ and ϕ(·) maps the set of branch feature maps to a single output tensor. A shallow residual connection further enhances the gradient stability and feature reuse. In HIDANet, aggregation is implemented via element-wise summation as in Equation 12


$$\begin{array}{l} \phi \left({\text{F}}_{1},\ldots ,{\text{F}}_{B}\right)=\overset{B}{\underset{b=1}{\sum }}{\text{F}}_{b} \end{array}$$ (12)

where *b* ∈ {1, …, *B*} indexes each branch.

##### 4.2.2.2 Convolution block attention module, CBAM

To refine discriminative features while suppressing background interference, CBAM is applied after multibranch fusion. Given ${\text{F}}_{iso}\in {\mathbb{R}}^{C_{out}\times H\times W}$, CBAM sequentially applies channel and spatial attention, where each attention map is applied via element-wise multiplication. The CBAM feature refinement process is described in Equation 13.


$$\begin{array}{l} {\text{F}}_{\text{cbam}}={\text{F}}_{\text{iso}}⊙{\text{M}}_{c}\left({\text{F}}_{\text{iso}}\right)⊙{\text{M}}_{s}({\text{F}}_{\text{iso}}⊙{\text{M}}_{c}\left({\text{F}}_{\text{iso}}\right)) \end{array}$$ (13)

where ⊙ denotes element-wise multiplication. The channel attention mechanism is computed as shown in Equation 14. First, the Channel Attention Module captures channel-wise importance by aggregating spatial information using global average pooling and global max pooling. These descriptors are passed through a shared multilayer perceptron (MLP) and combined via element-wise addition, followed by sigmoid activation:


$$\begin{array}{r} M_{c}(F_{\text{iso}})=\sigma \left(\text{MLP}(\text{AvgPool}(F_{\text{iso}})\right)\\ +\text{MLP}\left(\text{MaxPool}(F_{\text{iso}}))\right), \end{array}$$ (14)

where ${\text{M}}_{c}\in {\mathbb{R}}^{C\times 1\times 1}$. The channel-refined feature is obtained using Equation 15.


$$\begin{array}{l} {\text{F}}_{ca}={\text{F}}_{\text{iso}}⊙{\text{M}}_{c}\left({\text{F}}_{\text{iso}}\right), \end{array}$$ (15)

Next, the Spatial Attention Module emphasizes informative spatial regions by applying channel-wise pooling, followed by convolution. Specifically, the average and maximum projections across the channels are concatenated and passed through a 7 × 7 convolution as expressed in Equation 16.


$$\begin{array}{l} {\text{M}}_{s}\left({\text{F}}_{ca}\right)=\sigma \left(f^{7\times 7}\left(\left[\text{Avg}\left({\text{F}}_{ca}\right);\text{Max}\left({\text{F}}_{ca}\right)\right]\right)\right), \end{array}$$ (16)

where ${\text{M}}_{s}\in {\mathbb{R}}^{1\times H\times W}$. The spatially refined feature is obtained as in Equation 17.


$$\begin{array}{l} {\text{F}}_{sa}={\text{F}}_{ca}⊙{\text{M}}_{s}\left({\text{F}}_{ca}\right). \end{array}$$ (17)

The channel multilayer perceptron (MLP) adopts a bottleneck structure as in Equation 18:


$$\begin{array}{l} MLP\left(x\right)=W_{1}\left(\text{ReLU}\left(W_{0}\left(x\right)\right)\right), \end{array}$$ (18)

where *W*_0_:*C*→*C*/*r*, *W*_1_:*C*/*r*→*C*, and *r* ∈ 16 is the reduction ratio. Following attention refinement, the input feature **F**_iso_ is channel-wise concatenated with the attended output **F**_*sa*_, inspired by DenseNet-style connectivity is expressed in Equation 19.


$$\begin{array}{l} {\text{F}}_{\text{out}}=\left[{\text{F}}_{\text{iso}}∥{\text{F}}_{sa}\right],\;{\text{F}}_{\text{out}}\in {\mathbb{R}}^{\left(C+g\right)\times H\times W}, \end{array}$$ (19)

where [·∥·] denotes channel-wise concatenation and *g* is the growth rate. This inter-block dense connectivity, distinct from conventional element-wise residual addition, preserves all preceding feature representations and enables efficient feature reuse without spatial downsampling. Consequently, it enhances the gradient flow and mitigates information loss, which is critical for fine-grained larval feature extraction.

Across stacked ISOBlocks, the feature channels expand progressively (e.g., 16 → 32 → 64 → 128), forming a rich hierarchical representation while maintaining a compact parameter footprint.

#### 4.2.3 Feature aggregation and classification

After the final ISOBlock, global average pooling produces a compact feature vector as in Equation 20.


$$\begin{array}{l} {\text{F}}_{g}=\text{GAP}\left({\text{F}}_{sa}\right) \end{array}$$ (20)

The classifier applies two fully connected layers as defined in Equations 21 and 22 with Mish activation, as follows:


$$\begin{array}{l} \text{z}=\text{Mish}\left(W_{1}\left(\text{Dropout}\left({\text{F}}_{g}\right)\right)\right) \end{array}$$ (21)

$$\begin{array}{l} \hat{\text{y}}=W_{2}\left(\text{Dropout}\left(\text{z}\right)\right) \end{array}$$ (22)

where $W_{1}\in {\mathbb{R}}^{128\times 128}$ and $W_{2}\in {\mathbb{R}}^{7\times 128}$. The final class probabilities are obtained via softmax as in Equation 23.


$$\begin{array}{l} P\left(y_{i}|x\right)=\frac{\exp\left({ŷ}_{i}\right)}{\sum_{j=1}^{7}\exp\left({ŷ}_{j}\right)} \end{array}$$ (23)

Algorithm 1 demonstrates the workflow of the proposed HIDANet model, and Table 3 presents the layer-wise contribution of the proposed model. The proposed HIDANet architecture uses residual feature fusion and CBAM-based channel–spatial attention refinement with multibranch depthwise separable convolutions in the ISOBlocks, preserving spatial information. This is necessary for the morphological classification of post-larval underwater shrimp and facilitates successful multiscale feature learning of spatial regularities. The proposed network can be useful when monitoring aquaculture resources with limited resources, owing to its ability to apply the features efficiently and autonomously in terms of lighting and turbidity, with only 0.033M trainable parameters.

Algorithm 1 Proposed HIDANet for penaeidae post-larval (PL) classification. 
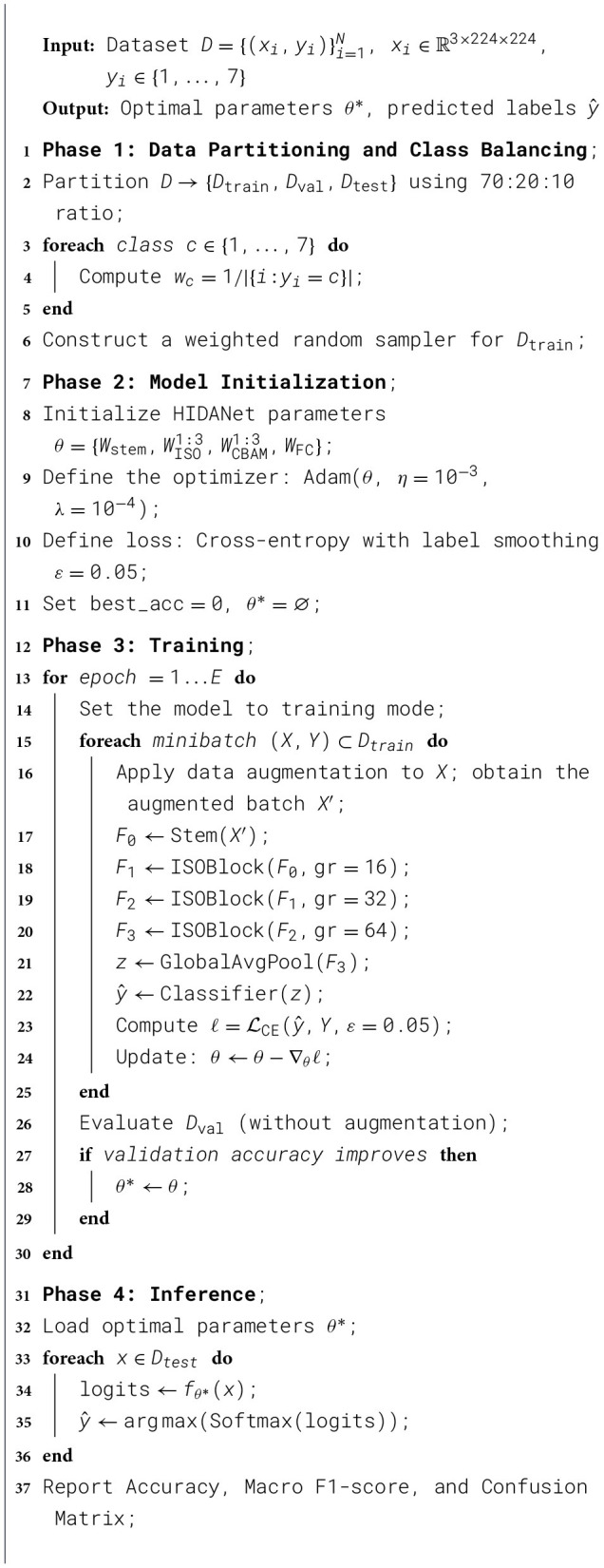

**Table 3:** Overview of proposed HIDANet architecture.

Stage	Main layer	Output shape	Params	Contribution
Stem	Conv2d + BN + Mish	16 × 112 × 112	464	Extracts primitive morphological cues such as body contour and appendage edges
ISOBlock-1	DSConv × 2 + CBAM	32 × 112 × 112	994	Captures fine-scale larval structural features, including rostrum and chromatophore patterns
ISOBlock-2	DSConv × 2 + CBAM	64 × 112 × 112	2,858	Encodes mid-level morphological features such as body segmentation and appendage development
ISOBlock-3	DSConv × 2 + CBAM	128 × 112 × 112	10,690	Learns stage-discriminative morphological representations across the PL5–PL12 developmental spectrum
Global pool	AdaptiveAvgPool + dropout	128	0	Aggregates morphological feature map into compact stage descriptor
Classifier	Linear + mish + dropout + linear	7	17,415	Maps morphological descriptors to seven post-larval stage predictions
Total			32,957	

### 4.3 Training dynamics and regularization strategy

To improve generalization and reduce overfitting, several regularization strategies have been incorporated into the HIDANet training pipeline, including strong data augmentation, class-balanced sampling, label smoothing, and adaptive learning rate scheduling.

To address the class imbalance, a weighted random sampler was used to construct balanced minibatches. For a class *c*_*i*_ containing *n*_*i*_ samples, the sampling probability was defined in Equation 24 as


$$\begin{array}{l} w_{i}=\frac{1}{n_{i}},\;P\left(c_{i}\right)=\frac{w_{i}}{\sum_{j}w_{j}} \end{array}$$ (24)

which increases the contribution of minority classes during optimization. The network was trained using categorical cross-entropy with label smoothing. For ground-truth label *y* ∈ {1, …, *C*} and logits *z* ∈ ℝ^*C*^, the loss, where *q*_*y*_ = 1−ε, *q*_*i*≠*y*_ = ε/*C*, and ε = 0.05 is defined in Equation 25 as


$$\begin{array}{l} L=-\overset{C}{\underset{i=1}{\sum }}q_{i}\log\left(p_{i}\right),\;p_{i}=\frac{e^{z_{i}}}{\sum_{j}e^{z_{j}}} \end{array}$$ (25)

Optimization was performed using the Adam optimizer (Kingma and Ba, 2014) with parameters β_1_ = 0.9, β_2_ = 0.999, and ϵ = 10^−8^. The initial learning rate was set to 1 × 10^−3^ with an L2 weight decay of 1 × 10^−4^. A ReduceLROnPlateau scheduler decreased the learning rate by a factor of 0.5 when validation accuracy plateaued over five epochs. Dropout (Srivastava et al., 2014) (*p* = 0.5) and batch normalization were applied to regularize the training further. The model was trained for up to 100 epochs with early stopping (Prechelt, 1998) (patience = 20), and the checkpoint with the highest validation accuracy was used for the final evaluation.

### 4.4 Background bias validation

Extensive testing was conducted in order to assess the dependency on the background color (Algorithm 2) to determine whether the HIDANet model relies on morphological characteristics rather than spurious relationships with background features. It is a significant validation exercise, particularly in the categorization of biological images, whereby the imaging environment and background can vary according to the developmental stage or mode of collection.

Algorithm 2 Background bias evaluation. 
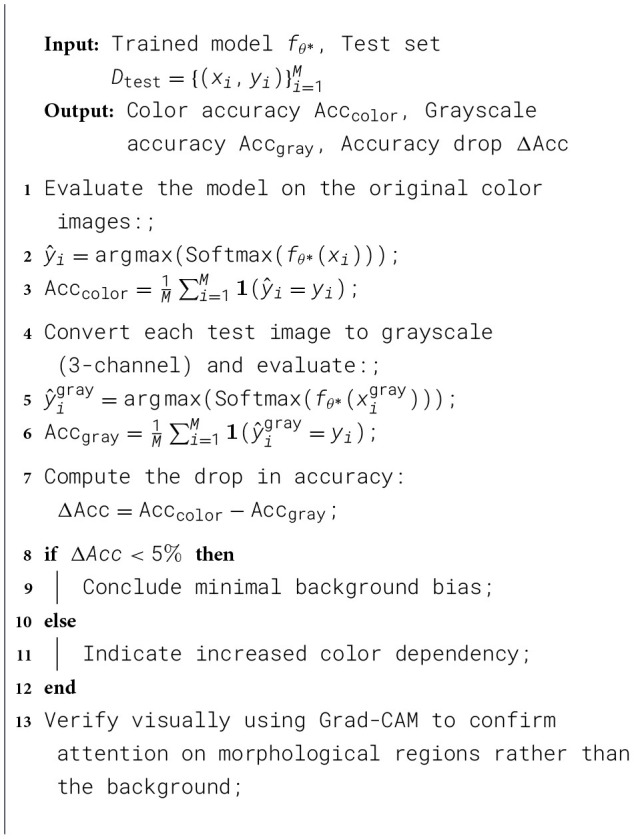

#### 4.4.1 Gray-scale robustness test

**Figure 5:**
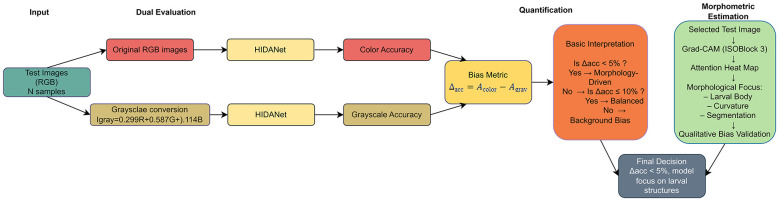
Background bias suppression pipeline for the proposed HIDANet model.

The primary validation methodology depicted in Figure 5 was to test the model on the test data, which were converted to grayscale to remove color information. The luminance-preserving transformation described in ITU-R Recommendation BT.601 (ITU-R 2011) was used to convert test photos to grayscale is given in Equation 26.


$$\begin{array}{l} {\text{I}}_{\text{gray}}=0.299R+0.587G+0.114B \end{array}$$ (26)

where *R*, *G*, and *B* represent the red, green, and blue channels, respectively. The grayscale intensity was replicated across the three channels to preserve compatibility with the network architecture. The classification accuracy on grayscale images (*A*_gray_) was compared with the accuracy obtained on the original color images (*A*_color_). The degree of color dependency was quantified using the following metric as defined in Equation 27.


$$\begin{array}{l} \Delta_{\text{acc}}=A_{\text{color}}-A_{\text{gray}} \end{array}$$ (27)

A degradation value of Δ_acc_ < 5% indicates minimal reliance on color cues and confirms robust morphology-based feature learning. Degradation values between 5% and 10% suggest balanced use of chromatic and structural information, whereas Δ_acc_>10% indicates significant color dependency, which may signal background-induced bias, a phenomenon widely reported in deep learning-based image classification (Geirhos et al., 2018).

#### 4.4.2 Gradient-weighted class activation mapping

To visualize the spatial regions contributing to the model's classification decisions, Gradient-weighted Class Activation Mapping (Grad-CAM) was employed. Grad-CAM generates class-specific localization maps by exploiting gradient information in the final convolutional layer. Let *A*^*k*^ ∈ ℝ^*H*×*W*^ denote the *k*-th feature map of the final convolutional layer. The importance weight $\alpha_{k}^{c}$ associated with class *c* is computed by performing global average pooling over the gradients as in Equation 28.


$$\begin{array}{l} \alpha_{k}^{c}=\frac{1}{HW}\overset{H}{\underset{i=1}{\sum }}\overset{W}{\underset{j=1}{\sum }}\frac{\partial y^{c}}{\partial A_{ij}^{k}} \end{array}$$ (28)

The resulting class-discriminative localization map $L_{\text{Grad-CAM}}^{c}\in {\mathbb{R}}^{H\times W}$ is obtained as in Equation 29.


$$\begin{array}{l} L_{\text{Grad-CAM}}^{c}=\text{ReLU}\left(\underset{k}{\sum }\alpha_{k}^{c}A^{k}\right) \end{array}$$ (29)

The ReLU operation ensures that only the features exerting a positive influence on the target class are retained. High-intensity regions in $L_{\text{Grad-CAM}}^{c}$ highlight spatial areas that most strongly drive the classification decisions. Grad-CAM visualizations were qualitatively analyzed to confirm whether the model attends to morphologically meaningful regions, such as shrimp body structures, larval segmentation, and pigmentation patterns, rather than irrelevant background areas. Such attention patterns provide interpretability and support the effectiveness of the proposed background bias mitigation strategy.

### 4.5 Integrated stage prediction and morphometric analysis framework

Test images underwent identical preprocessing as the validation data, including bilinear resizing to 224 × 224, normalization to [0, 1], and standardization using ImageNet statistics **μ** = [0.485, 0.456, 0.406] and **σ** = [0.229, 0.224, 0.225]. The standardized tensor *X* ∈ ℝ^3 × 224 × 224^ was forwarded through the trained network θ^*^ to produce class probabilities as in Equation 30.


$$\begin{array}{l} P\left(y=c|X\right)=\frac{\exp\left({ŷ}_{c}\right)}{\sum_{j=1}^{C}\exp\left({ŷ}_{j}\right)},\;c\in \left\{1,\ldots ,C\right\} \end{array}$$ (30)

where *C* = 7 denotes the number of developmental stage classes. The predicted development stage was obtained as in Equation 31.


$$\begin{array}{l} ŷ=\arg\underset{c}{\max}\;P\left(y=c|X\right) \end{array}$$ (31)

with prediction confidence defined as in Equation 32.


$$\begin{array}{l} C_{\text{conf}}=\underset{c}{\max}\;P\left(y=c|X\right)\times 100\%. \end{array}$$ (32)

#### 4.5.1 Developmental stage prediction

To complement the stage prediction, an automated larvae counting and morphometric analysis algorithm was applied directly to the microscopy images. RGB images were first converted to grayscale luminance, *G*. Contrast enhancement was performed using Contrast Limited Adaptive Histogram Equalization (CLAHE) (Zuiderveld, 1994; Sugunapriya and Markkandan, 2024) with a clip limit λ = 2.0 and a tile size 8 × 8. Foreground–background separation was achieved using adaptive Gaussian thresholding as expressed in Equation 33 (Bradley and Roth, 2007),


$$B(x,y)=\{\begin{array}{ll} 1, & G_{\text{enh}}(x,y)<T(x,y)\\ 0, & \text{otherwise} \end{array}$$ (33)

where *T*(*x, y*) denotes the locally computed Gaussian-weighted threshold with offset constant *C* = 3.

Morphological refinement was performed using elliptical structuring elements as shown in Equation 34


$$\begin{array}{l} B_{\text{open}}=\left(B⊖S_{2}\right)⊕S_{2},\;B_{\text{refined}}=\left(B_{\text{open}}⊕S_{3}\right)⊖S_{3} \end{array}$$ (34)

where *S*_2_ and *S*_3_ denote 2 × 2 and 3 × 3 elliptical kernels, respectively.

#### 4.5.2 Geometric morphometric filtering

External contours {*C*_*i*_} were extracted from the refined mask and filtered using geometric constraints to suppress debris and artifacts. Valid larvae satisfied the area constraint defined in Equation 35.


$$\begin{array}{l} 20<A_{i}<3000j \end{array}$$ (35)

where *A*_*i*_ denotes the contour area of the pixels. To quantify elongation, skeletonization (Zhang and Suen, 1984) was applied to each candidate region to obtain a one-pixel-wide medial axis representation *S*_*i*_. Skeleton length was estimated as as shown in Equation 36.


$$\begin{array}{l} L_{i}=\underset{\left(x,y\right)\in S_{i}}{\sum }1 \end{array}$$ (36)

Contours satisfying *L*_*i*_≥15 and the solidity condition defined in Equation 37 were retained for further analysis,


$$\begin{array}{l} \sigma_{i}=\frac{A_{i}}{\text{Area}\left(\text{ConvexHull}\left(C_{i}\right)\right)}\geq 0.15 \end{array}$$ (37)

were considered valid.

The final larvae count was computed as in Equation 38.


$$\begin{array}{l} N=|\left\{C_{i}:20<A_{i}<3000\wedge L_{i}\geq 15\wedge \sigma_{i}\geq 0.15\right\}| \end{array}$$ (38)

**Figure 6:**
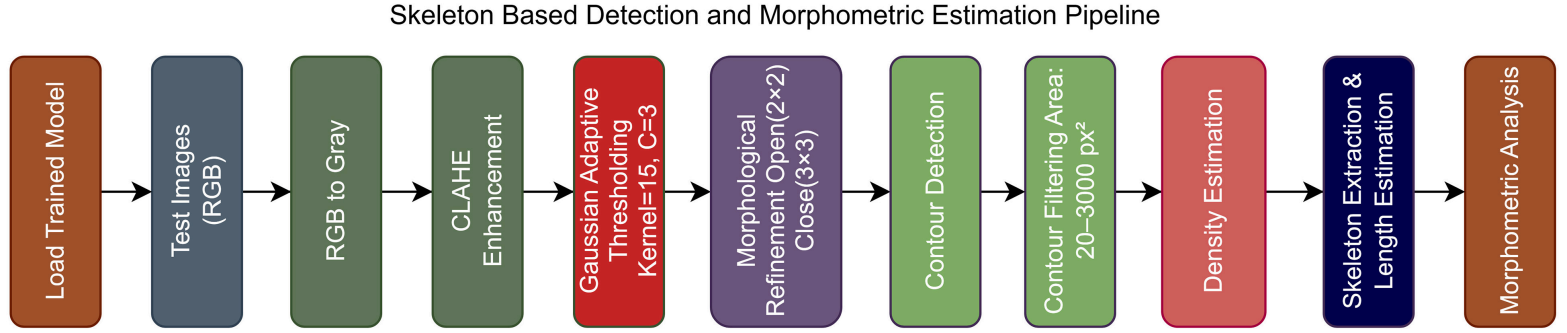
Post-training analysis workflow for automated larvae counting and morphological measurement.

Population morphometric statistics were computed as shown in Equation 39


$$\begin{array}{l} \mu_{A}=\frac{1}{N}\overset{N}{\underset{i=1}{\sum }}A_{i} \end{array}$$ (39)

where, mean area, μ_*A*_, median area Med_*A*_, standard deviation σ_*A*_, mean skeleton length $\bar{L}$, and median skeleton length. Larval density was categorized as: low if *N* < 50, Medium if 50 ≤ *N* < 150, and High if *N*≥150. Size classification based on median area was defined as Small if Med_*A*_ < 50, Medium if 50 ≤ Med_*A*_ < 150, and Large if Med_*A*_≥150. This unified framework enables simultaneous classification and population density estimation of digital larval images, supporting future deployment in an automated aquaculture production environment. Algorithm 3 and Figure 6 demonstrate the method used to identify and estimate the morphometric characteristics of detected PL.

Algorithm 3 Unified stage classification and larvae count estimation. 
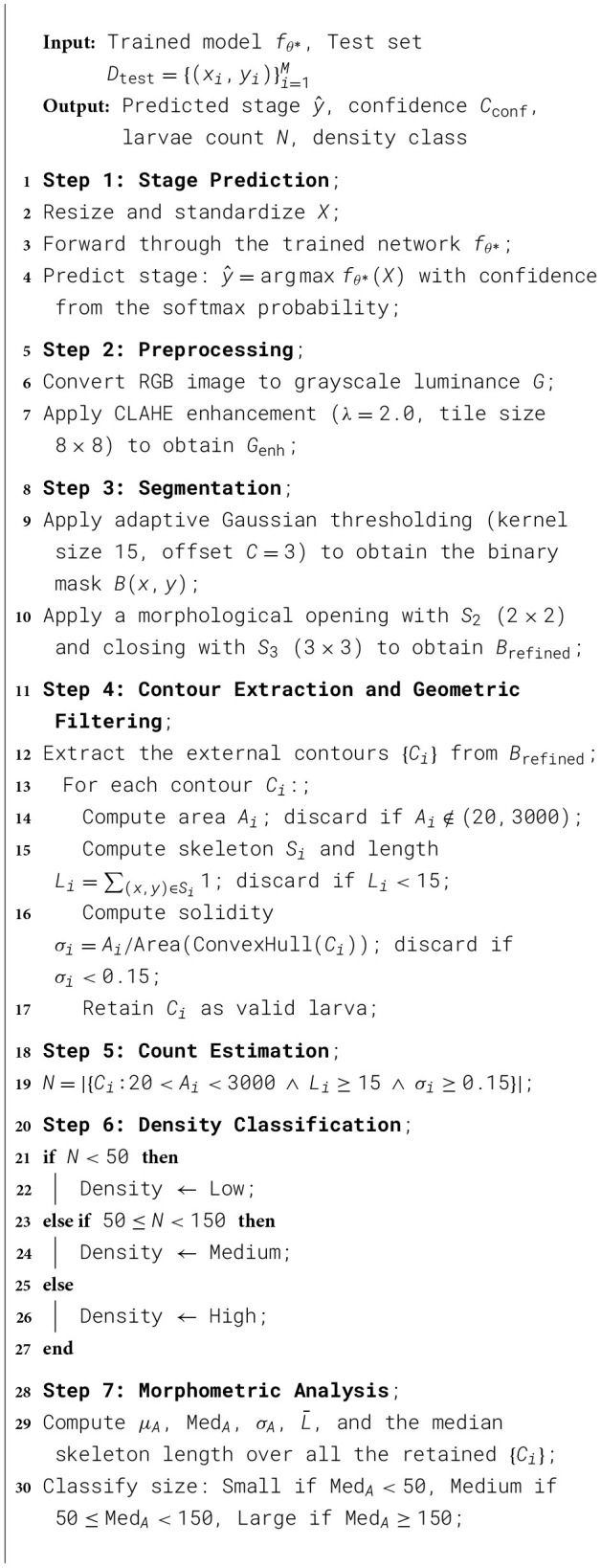

## 5 Experimental setup and evaluation metrics

### 5.1 Hardware/software environment

The detailed specifications of the hardware, software, and model hyperparameters are summarized in Table 4 to ensure transparency and reproducibility of the experimental setup. The training configuration and parameters described in Section 4.3 were executed in this environment to maintain consistency in all the experiments. In addition, all baseline models employed for model comparison were implemented and trained from scratch under identical experimental conditions, including the same dataset splits, optimizer, learning rate schedule, augmentation pipeline, and early stopping criteria. To ensure a fair and unbiased comparison, pre-trained ImageNet weights were not used for any model.

**Table 4:** Experimental setup of proposed HIDANet model.

Hardware environment	Software environment	Model hyperparameters
GPU model: NVIDIA L4 (23 GB)	Python 3.12.12	Batch size: 32
CUDA available: Yes	PyTorch 2.10.0 (+ cu128)	Loss function: Cross-Entropy with label smoothing (ε = 0.05)
CUDA version: 12.8	TensorFlow 2.19.0	Optimizer: Adam (*lr* = 1 × 10^−3^, β_1_ = 0.9, β_2_ = 0.999, weight decay = 1 × 10^−4^)
Driver/CUDA runtime: 550.54.15/CUDA 12.4	NumPy 2.0.2	Learning rate scheduler: ReduceLROnPlateau (factor = 0.5, patience = 5)
cuDNN version: 9.1.0.2	scikit-learn 1.6.1	Augmentation: Color jitter (brightness, contrast, saturation = 0.5; hue = 0.3), Random grayscale conversion (probability = 0.3), Random autocontrast (probability = 0.2)
GPU count: 1	Matplotlib 3.10.0	Sampling strategy: WeightedRandomSampler for class imbalance
VRAM utilization: 5.3/22 GB (idle)	Pandas 2.2.2	Epochs: 100 (with early stopping)
CPU/RAM: Intel Xeon (2 vCPUs, 53 GB RAM)	OS: Ubuntu 20.04 (Linux 6.6.105+)	—

### 5.2 Cross-validation and evaluation metrics

In order to guarantee a sound performance estimation and reduce overfitting, a stratified three-fold cross-validation plan was implemented on the training subset. The 3-fold configuration was selected to ensure sufficient samples per fold for minority classes under the class-imbalanced distribution. All hyperparameters were fixed a priori based on preliminary experiments and were not tuned within the cross-validation loop. The cross-validation was only done on the 70% training, but the validation and test sets were not seen at all in this phase to avoid data leakage.

This was performed by initializing each fold of the model with random weights and training it separately. The subset that was used for optimization was approximately two-thirds of the training subset, and internal validation was one-third of the training subset. Each fold was computed to calculate the performance metrics, and the results are presented as the mean and standard deviation to measure stability across partitions. Once cross-validation was performed, the last model was again trained with the full training set and then checked against the held-out validation and test sets to report the final performance.

Three complementary perspectives of analysis were used to assess the model evaluation: (i) classification performance with class imbalance, (ii) ability to withstand color suppression to measure the level of background bias, and (iii) quality of feature representation analysis.

#### 5.2.1 Classification performance under class imbalance

To evaluate the predictive performance across shrimp post-larval (PL) stages, Accuracy, Macro-F1, Matthews Correlation Coefficient (MCC), and Cohens Kappa (κ) (Cohen, 1960) were computed. Accuracy was calculated as using Equation 40.


$$\begin{array}{l} \text{Accuracy}=\frac{TP+TN}{TP+TN+FP+FN} \end{array}$$ (40)

where *TP* denotes true positives, *TN* denotes true negatives, *FP* denotes false positives, and *FN* denotes false negatives. Although accuracy provides an overall performance measure, it may be influenced by class imbalances. To address imbalance across PL stages, Macro-F1 was computed using Equation 41. as the arithmetic mean of the per-class F1-scores:


$$\begin{array}{l} \text{Macro-F1}=\frac{1}{C}\overset{C}{\underset{i=1}{\sum }}F1_{i} \end{array}$$ (41)

where *C* represents the total number of PL stages, and *F*1_*i*_ is the F1-score of class *i*. This metric assigns equal importance to each stage of PL. The Matthews Correlation Coefficient (MCC) (Chicco and Jurman, 2020) was used as a robust correlation-based metric derived from the confusion matrix. The MCC provides a balanced evaluation, even when class distributions are unequal.

Cohens Kappa (κ) is defined as in Equation 42.


$$\begin{array}{l} \kappa =\frac{p_{o}-p_{e}}{1-p_{e}} \end{array}$$ (42)

*p*_*o*_ denotes the observed agreement, and *p*_*e*_ denotes the agreement expected by chance. Kappa measures the reliability of the classification achieved by the model, beyond random agreement.

#### 5.2.2 Robustness to color suppression

The models were used to test the dependence on color cues in grayscale-transformed images. Robustness was measured by the accuracy drop and MCC drop as in Equations 43 and 44; the greater the performance degradation, the lower the robustness.


$$\begin{array}{l} \text{Accuracy Drop}={\text{Accuracy}}_{\left(\text{RGB}\right)}-{\text{Accuracy}}_{\left(\text{Gray}\right)} \end{array}$$ (43)

$$\begin{array}{l} \text{MCC Drop}={\text{MCC}}_{\left(\text{RGB}\right)}-{\text{MCC}}_{\left(\text{Gray}\right)} \end{array}$$ (44)

Two cosine similarities in the learned embedding space were calculated as in Equation 45 in order to understand the vulnerability of the internal feature level to the cutoff of color information. Color–gray cosine similarity calculates the mean similarity of color and gray feature vectors at the sample level using Equation 46:


$$\begin{array}{l} \text{CosSim}\left(i\right)=\frac{{\text{F}}_{\text{color}}^{\left(i\right)}\cdot {\text{F}}_{\text{gray}}^{\left(i\right)}}{||{\text{F}}_{\text{color}}^{\left(i\right)}||\cdot ||{\text{F}}_{\text{gray}}^{\left(i\right)}||} \end{array}$$ (45)

$$\begin{array}{l} {\text{CosSim}}_{\text{avg}}=\frac{1}{N}\overset{N}{\underset{i=1}{\sum }}\text{CosSim}\left(i\right) \end{array}$$ (46)

where ${\text{F}}_{color}^{\left(i\right)}$ and ${\text{F}}_{gray}^{\left(i\right)}$ are the feature vectors extracted from the color and grayscale versions of sample *i*, respectively, and *N* is the total number of test samples. The fact that the suppression of color information has minimal influence on the learned features suggests that the model is based on morphological features instead of background scenes, with a value of approximately 1.0.

In order to evaluate the class-level robustness, the Mean Centroid Cosine Similarity was used to compare the class-average feature vectors (centroids) representing color and grayscale images as defined in Equation 47.


$$\begin{array}{l} \mu_{c}^{\text{color}}=\frac{1}{\left|S_{c}\right|}\underset{i\in S_{c}}{\sum }{\text{F}}_{\text{color}}^{\left(i\right)},\;\mu_{c}^{\text{gray}}=\frac{1}{\left|S_{c}\right|}\underset{i\in S_{c}}{\sum }{\text{F}}_{\text{gray}}^{\left(i\right)} \end{array}$$ (47)

The centroid cosine similarity between the color and grayscale feature centroids was calculated using Equation 48


$$\begin{array}{l} {\text{CentroidSim}}_{c}=\frac{\mu_{c}^{\text{color}}\cdot \mu_{c}^{\text{gray}}}{||\mu_{c}^{\text{color}}||\cdot ||\mu_{c}^{\text{gray}}||} \end{array}$$ (48)

The overall mean centroid cosine similarity was computed using Equation 49.


$$\begin{array}{l} {\text{CentroidSim}}_{\text{avg}}=\frac{1}{C}\overset{C}{\underset{c=1}{\sum }}{\text{CentroidSim}}_{c} \end{array}$$ (49)

where *S*_*c*_ represents the collection of samples belonging to class *c* and *C* represents the total number of PL stages. To the extent that the color information is suppressed, high centroid similarities show that the class-level feature spaces are invariant, and the model is therefore based on morphological and structural features other than chromatic features.

#### 5.2.3 Feature representation quality

To analyze the structural organization of the learned embeddings, clustering-based metrics were computed for high-dimensional feature representations. The Adjusted Rand Index (ARI) was used to measure the similarity between predicted clusters and true PL stage labels, correcting for chance agreement. Normalized Mutual Information (NMI) quantifies the shared information between cluster assignments and ground-truth labels based on information theory.

Silhouette Score (Rousseeuw, 1987) was calculated as in Equation 50,


$$\begin{array}{l} s\left(i\right)=\frac{b\left(i\right)-a\left(i\right)}{\max\left(a\left(i\right),b\left(i\right)\right)} \end{array}$$ (50)

where *a*(*i*) represents the mean intracluster distance and *b*(*i*) represents the mean nearest-cluster distance. This metric evaluates the compactness of a cluster and its separation. t-SNE visualization (van der Maaten and Hinton, 2008) was used to qualitatively inspect the distributions of the clusters.

## 6 Results

### 6.1 Cross-validation and overall proposed model performance

**Figure 7:**
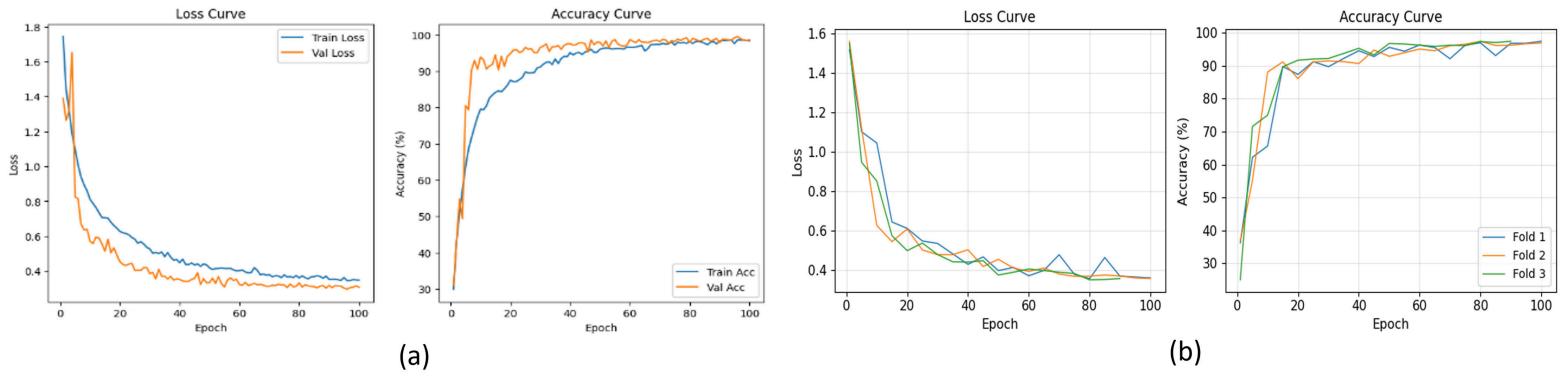
Training performance of **(a)** proposed HIDANet and **(b)** stratified k-fold cross-validation.

**Figure 8:**
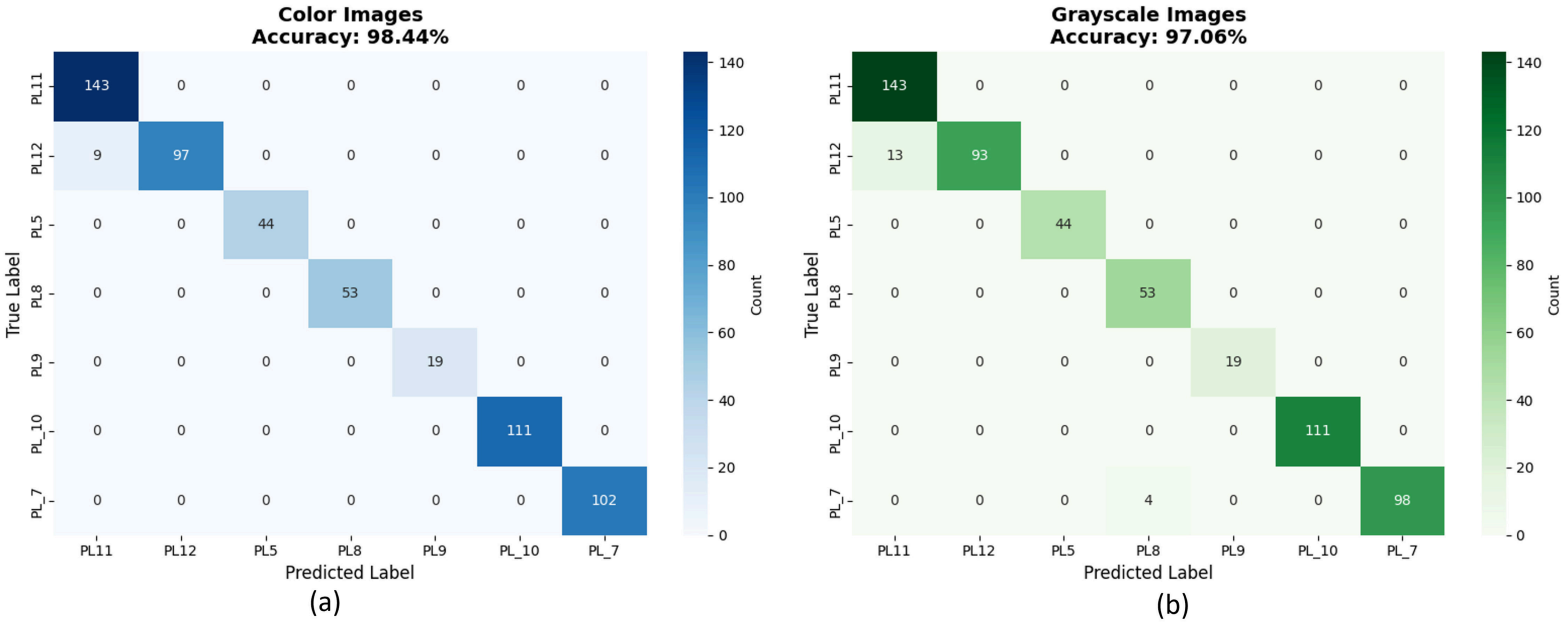
Confusion matrix of **(a)** RGB and **(b)** grayscale test images.

The proposed HIDANet architecture demonstrated stable and consistent performance under the threefold stratified cross-validation protocol. Across the three folds, the model achieved a mean validation accuracy of 97.77% ± 0.43%, indicating low variability between independent training partitions (Table 5). The individual fold accuracies ranged from 97.43% to 98.38%, demonstrating stable convergence and limited sensitivity to sample partitioning. Based on the quantitative performance analysis, the training log shown in Figure 7, and the confusion matrix, Figure 8 depicts the performance of the classification task across the seven shrimp post-larval (PL) stages, where the elements correspond to correct classifications and the off-diagonal elements indicate misclassification occurrences. Unless otherwise stated, all evaluation metrics, including precision, recall, and F1-score, were computed on the RGB test set (*n* = 578). The classification accuracy was additionally reported under grayscale evaluation as part of the background bias validation framework.

**Table 5:** Three-fold cross-validation (training subset) and held-out sets performance of HIDANet.

Cross-validation		Final evaluation (held-out sets)		
Fold	Val. acc. (%)	Metric	Val. set	Test set
Fold 1	98.38	Accuracy (%)	99.49	98.44
Fold 2	97.43	Precision (macro)	0.99	0.985
Fold 3	97.50	Recall (macro)	0.98	0.984
**Mean** **±SD**	**97.77** **±0.43**	F1-score (macro)	0.99	0.984

Bold values indicate the mean and standard deviation obtained from the three-fold cross-validation.

**Figure 9:**
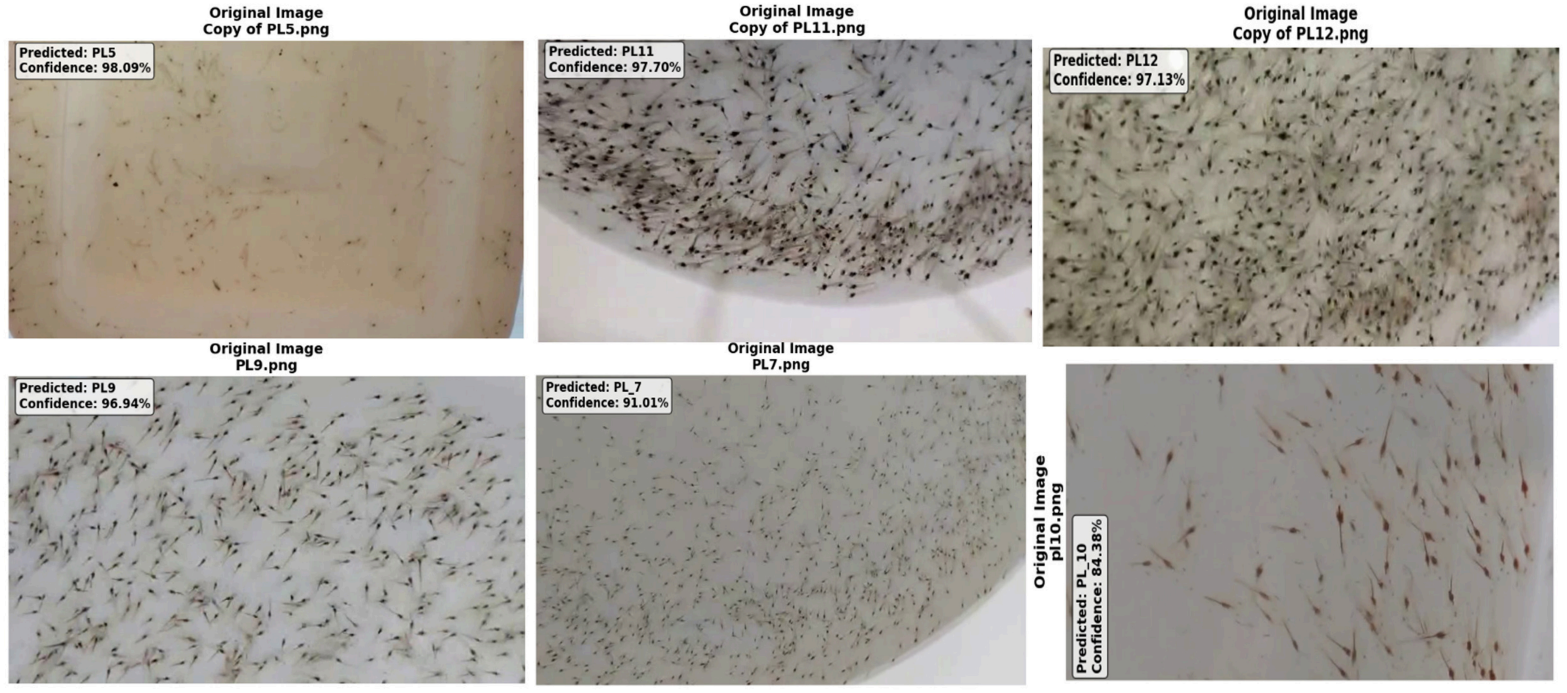
Visualization of predicted test images of various PL stages.

The misclassification was restricted solely to the PL11–PL12 boundary, where nine PL12 samples were misclassified as PL11, while all other stages were discriminated perfectly. This confusion is biologically grounded in the PL11–PL12 transition; rostrum elongation remains incomplete, uropod differentiation has not yet reached a distinguishable form, and chromatophore distribution patterns remain spatially similar between the two stages. These overlapping morphological expressions make the PL11–PL12 boundary the most subtle transition in the PL5–PL12 developmental sequence. Grad-CAM attention maps in Section 6.4 further confirmed this interpretation, where the misclassified PL12 sample showed weak and diffuse activation, indicating insufficient detection of PL12-discriminative features at this transitional boundary. Figure 9 demonstrates that the strong multiclass separation can be achieved on unknown test samples.

### 6.2 Per class performance across PL stages

#### 6.2.1 Per-class performance analysis of proposed HIDANet

The proposed HIDANET achieved a consistently high per-class cross-validation performance across all developmental stages, as shown in Tables 6, 7. The mean cross-validation accuracy in the majority of the PL stages was above 97%. The mean accuracy of PL12 was relatively low (91.64% ± 3.02%), which represents morphological similarity to the next stages, particularly PL11. Table 5 shows that the test set performance was consistent with the cross-validation results, confirming the stability of the proposed model.

**Table 6:** Per-class cross-validation accuracy (mean ± SD) and final test.

Metric	PL5	PL7	PL8	PL9	PL10	PL11	PL12
CV mean accuracy (%)	100.0	97.9	99.3	98.0	99.4	99.1	91.6
CV standard deviation (%)	0.0	0.38	0.99	1.63	0.46	0.73	3.02
Independent test accuracy (%)	100	100	100	100	100	100	91.5

**Table 7:** Per-class RGB and grayscale accuracy (%) across augmentation strategies.

Aug.	Metric	PL11	PL12	PL5	PL8	PL9	PL10	PL7
Baseline	RGB Acc.	100.0	100.0	100.0	100.0	100.0	100.0	100.0
	Gray Acc.	99.3	23.6	0.0	100.0	100.0	98.2	72.5
	Drop (%)	0.7	76.4	100.0	0.0	0.0	1.8	27.5
	Recall	1.00	1.00	1.00	1.00	1.00	1.00	1.00
Moderate	RGB Acc.	100.0	100.0	100.0	100.0	100.0	99.1	99.0
	Gray Acc.	68.5	99.1	0.0	26.4	100.0	38.7	21.6
	Drop (%)	31.5	0.9	100.0	73.6	0.0	60.4	77.5
	Recall	1.00	1.00	1.00	1.00	1.00	0.90	0.90
Strong	RGB Acc.	100.0	91.5	100.0	100.0	100.0	100.0	100.0
	Gray Acc.	100.0	87.7	100.0	100.0	100.0	100.0	96.1
	Drop (%)	0.0	3.8	0.0	0.0	0.0	0.0	3.9
	Recall	1.00	0.90	1.00	1.00	1.00	1.00	0.90

**Figure 10:**
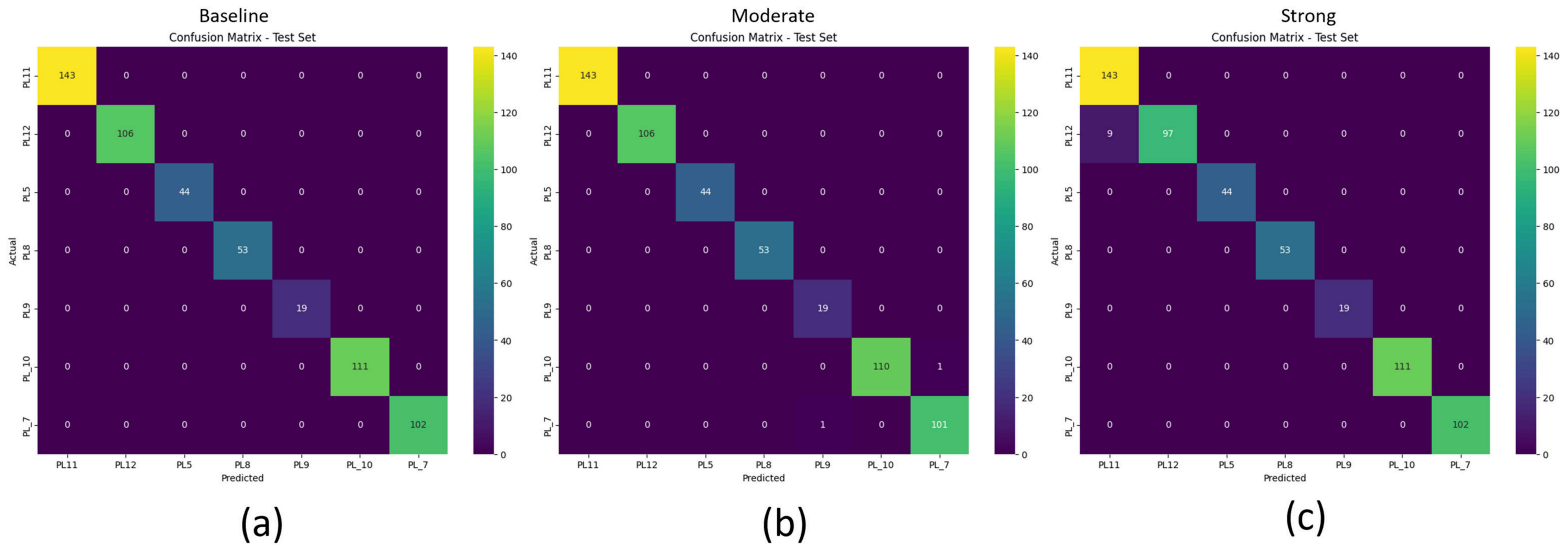
Confusion matrices of HIDANet under **(a)** baseline, **(b)** moderate, and **(c)** strong augmentation.

The baseline configuration is characterized by very strong class-specific grayscale collapses, particularly for PL5 (100% absolute collapse), PL12 (76.4% collapse), and PL7 (27.5% collapse), which indicates a strong dependence on color information. For some classes, moderate augmentation showed stable results but fluctuating results, and PL5, PL8, PL10, and PL7 showed catastrophic drops. However, robust bias control augmentation showed stable grayscale for all classes, and the maximum values for degradation were no more than 4%, thus showing good resistance to class-level shortcut learning. From the confusion matrix, Figure 10 shows strong diagonal dominance across the baseline, moderate, and strong augmentation.

Per-class recall demonstrated strong performance across all PL stages. As the augmentation level was high, a slight drop was noticed in PL12 (recall = 0.90), but for all other classes, the recall was close to perfect. Although the dataset is class-imbalanced, there was no systematic drop in performance levels between stages. The baseline model had the highest accuracy; it was highly affected by the suppression of color channels. Aggressive augmentation caused a slight drop in the peak accuracy, a significant boost in the robustness and consistency of features, and a high degree of color invariance. The above results show that aggressive augmentation is a more reliable technique for the classification of shrimp PL stages.

#### 6.2.2 Per-class performance and statistical reliability

To evaluate class-wise performance and statistical reliability, per-class accuracy along with bootstrap and Wilson 95% confidence intervals (CI) (Efron and Tibshirani, 1994; Brown et al., 2001) were computed on the test set (*n* = 578), as summarized in Table 8. The proposed HIDANet achieved perfect classification (100%) for six out of seven classes (PL11, PL5, PL8, PL9, PL10, and PL7). For these classes, the bootstrap confidence intervals collapse to [100%, 100%] as all samples are correctly classified. However, the corresponding Wilson confidence intervals provide more informative lower bounds by accounting for finite sample uncertainty.

**Table 8:** Per-class accuracy with Bootstrap and Wilson 95% confidence intervals for HIDANet (proposed) (*n* = 578).

Class	N	Accuracy (%)	Bootstrap CI (95%)	Wilson CI (95%)	Note
PL11	143	100.00	[100.00%, 100.00%]	[97.38%, 100.00%]	Perfect classification
PL12	106	91.51	[85.85%, 96.23%]	[84.65%, 95.47%]	Lowest accuracy; wide CI
PL5	44	100.00	[100.00%, 100.00%]	[91.97%, 100.00%]	Perfect classification
PL8	53	100.00	[100.00%, 100.00%]	[93.24%, 100.00%]	Perfect classification
PL9	19	100.00	[100.00%, 100.00%]	[83.18%, 100.00%]	Minority class; Wilson CI reported
PL10	111	100.00	[100.00%, 100.00%]	[96.65%, 100.00%]	Perfect classification
PL7	102	100.00	[100.00%, 100.00%]	[96.37%, 100.00%]	Perfect classification

For instance, PL9 (*n* = 19), despite achieving 100% accuracy, exhibited a wider Wilson interval [83.18%, 100%], reflecting the limited sample size. In contrast, PL12 is the only class with imperfect performance, achieving an accuracy of 91.51%, with a relatively wider bootstrap CI [85.85%, 96.23%] and Wilson CI [84.65%, 95.47%], indicating higher variability and increased classification difficulty.

The class distribution in the test set is moderately imbalanced, with PL11 (24.7%) and PL10 (19.2%) forming the majority classes, whereas PL9 represents a minority class (3.3%). Despite this imbalance, the model maintained robust performance across all classes, including the minority class, demonstrating effective generalization and reduced bias.

#### 6.2.3 Model calibration analysis

To assess the reliability of predicted confidence scores in a hatchery quality control context, the Expected Calibration Error (ECE) was computed on the test set (*N* = 578) using a 10-bin reliability analysis, following standard calibration evaluation practices (Guo et al., 2017). The model achieved an ECE of 0.0269, which indicates a well-calibrated prediction. As shown in Table 9, 92.7% of the test samples (536/578) fall within the highest confidence bin (0.91.0), achieving perfect accuracy in that bin.

**Table 9:** Calibration bin analysis of HIDANet predictions.

Confidence bin	N	Avg. confidence	Avg. accuracy	Gap
0.4–0.5	5	0.4765	0.4000	0.0765
0.5–0.6	5	0.5523	0.6000	0.0477
0.6–0.7	4	0.6527	0.5000	0.1527
0.7–0.8	11	0.7439	0.9091	0.1652
0.8–0.9	17	0.8567	0.8824	0.0257
0.9–1.0	536	0.9775	1.0000	0.0225
Overall	578	0.9593	0.9827	ECE = 0.0269, MCE = 0.1652

The Maximum Calibration Error (MCE) of 0.1652 is observed in the 0.70.8 confidence bin (*n* = 11), reflecting underconfidence rather than overconfidence. Specifically, the model predicts confidence levels between 70% and 80% while achieving an actual accuracy of 90.9% in this bin, which does not pose a risk in safety-critical deployment scenarios.

Per-class analysis (Table 10) shows that five out of seven classes achieve 100% accuracy, with a mean confidence exceeding 0.96. In contrast, PL12 exhibits the lowest mean confidence (0.8743) and lowest accuracy (91.51%), indicating that the model appropriately expresses uncertainty for more challenging samples rather than masking errors.

**Table 10:** Per-class confidence statistics for HIDANet predictions.

Class	N	Mean Conf.	Min	Max	Accuracy
PL5	35	0.9684	0.9306	0.9821	100.00%
PL_7	110	0.9666	0.7158	0.9913	100.00%
PL8	63	0.9860	0.9357	0.9954	100.00%
PL9	17	0.9873	0.9617	0.9970	100.00%
PL_10	106	0.9758	0.0604	0.9951	99.06%
PL11	141	0.9640	0.7275	0.9929	100.00%
PL12	106	0.8743	0.1301	0.9967	91.51%
Overall	578	0.9593	–	–	98.27%

HIDANet achieved a test accuracy of 98.44% based on hard classification decisions. The 10-bin calibration analysis yields a bin-weighted average accuracy of 98.27% and an overall mean confidence of 0.9593, producing an Expected Calibration Error (ECE) of 0.0269 and a Maximum Calibration Error (MCE) of 0.1652. The small ECE confirms that HIDANet does not exhibit the overconfidence commonly reported in deep learning classifiers.

### 6.3 Background bias analysis

From Tables 5, 11, the baseline model achieved perfect RGB test accuracy (1.000), Cohen's κ (1.000), Macro-F1 (1.000), and MCC (1.000) on color inputs. The moderate augmentation strategy maintained high RGB performance (accuracy = 0.9965, κ = 0.9958, and MCC = 0.9958) with improved generalization, whereas the strong augmentation strategy recorded a modest accuracy decline (accuracy = 0.9844) and Macro-F1 (0.98), with Cohen's κ = 0.9810 and MCC remaining high (0.9813), confirming stable multiclass predictive performance.

**Table 11:** Effect of augmentation on model performance and color robustness at 100 epochs.

Augmentation	RGB acc.	RGB κ	RGB MCC	Gray acc.	Gray κ	Gray MCC	Acc. drop ↓	κ drop ↓	MCC drop ↓
Baseline	**1.0000**	**1.0000**	**1.0000**	0.6626	0.5873	0.5974	0.3374	0.4127	0.4026
Moderate	0.9965	0.9958	0.9958	0.7595	0.7056	0.7230	0.2370	0.2901	0.2728
Strong (proposed)	0.9844	0.9810	0.9813	**0.9689**	**0.9620**	**0.9628**	**0.0156**	**0.0190**	**0.0185**

Bold values indicate the best-performing result in each comparison.

Grayscale evaluation revealed the extent of background color dependency across configurations. The baseline model suffered a dramatic performance collapse (accuracy drop = 0.3374, gray κ = 0.5873, and MCC drop = 0.4026), confirming heavy reliance on chromatic cues. Moderate augmentation partially mitigated this (accuracy drop = 0.2370, gray κ = 0.7056, and MCC drop = 0.2728), while the strong augmentation model exhibited minimal grayscale degradation (accuracy drop = 0.0156, gray κ = 0.9620, and MCC drop = 0.0185), demonstrating successful morphology-driven, color-invariant feature learning.

### 6.4 Feature space structure

**Figure 11:**
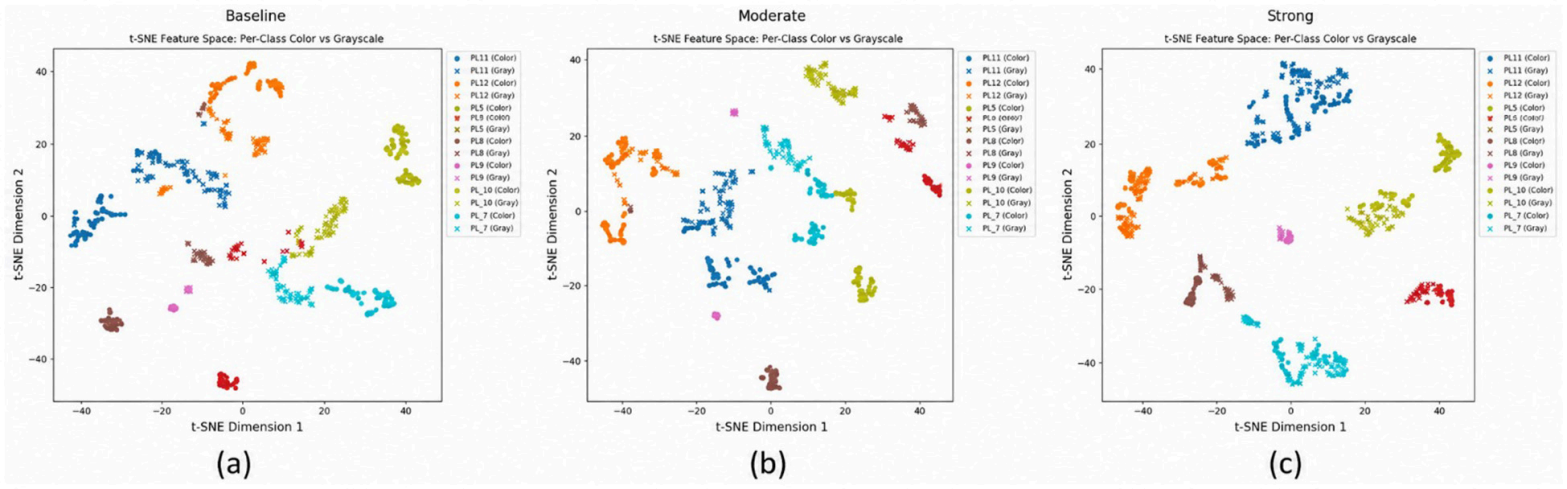
t-SNE feature space comparison of **(a)** baseline, **(b)** moderate, and **(c)** strong augmentation strategies in color vs. grayscale.

**Figure 12:**
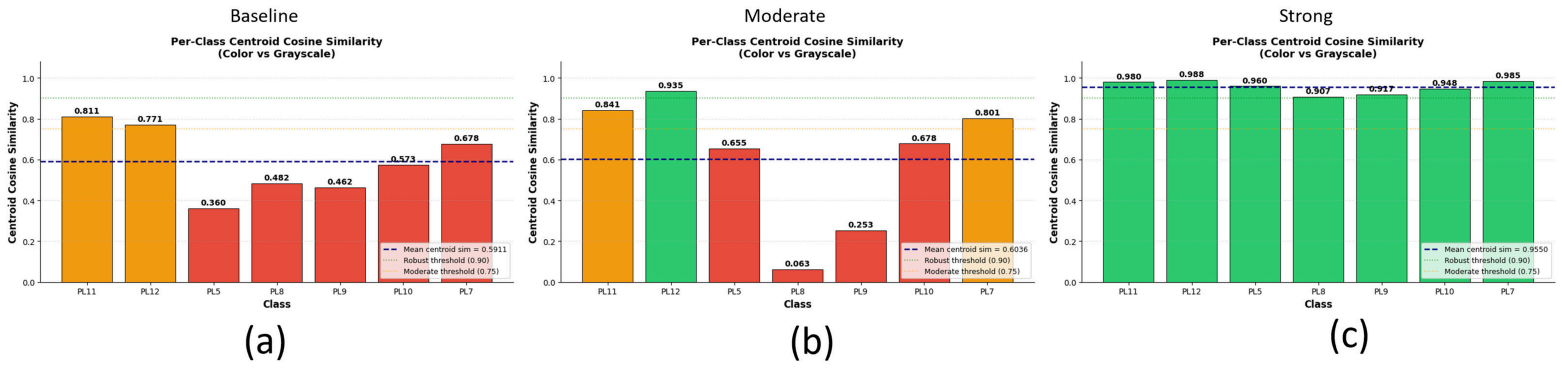
Per-class centroid cosine similarity for different augmentation strategies: **(a)** Baseline augmentation, **(b)** Moderate augmentation, and **(c)** Strong augmentation.

t-SNE feature space visualizations (Figure 11) further confirmed the progressive improvement in class separability and color–grayscale alignment across augmentation strategies. The feature clustering analysis presented in Table 12 further supports these findings. Strong augmentation achieved the highest ARI (0.945) and NMI (0.948), demonstrating an improved alignment between the learned feature representations and true class labels. Although the baseline model showed a higher Silhouette score (0.446), its pronounced grayscale performance drop suggests that clustering may be influenced by color-based separability rather than the intrinsic morphological structure. Moderate augmentation yielded comparatively lower ARI (0.726) and silhouette (0.253), indicating less compact feature organization. Figure 12, with per-class centroid cosine similarity, highlights that the strong augmentation strategy produces more color-invariant feature representations across all PL stages.

**Table 12:** Feature space structure and color invariance analysis.

Augmentation	ARI	NMI	Silhouette	Mean centroid cosine	Color–gray cosine similarity
Baseline	0.906	0.926	**0.446**	0.5911	0.61
Moderate	0.726	0.853	0.253	0.6521	0.65
Strong	**0.945**	**0.948**	0.383	**0.9596**	**0.96**

Bold values indicate the best-performing result in each comparison.

**Figure 13:**
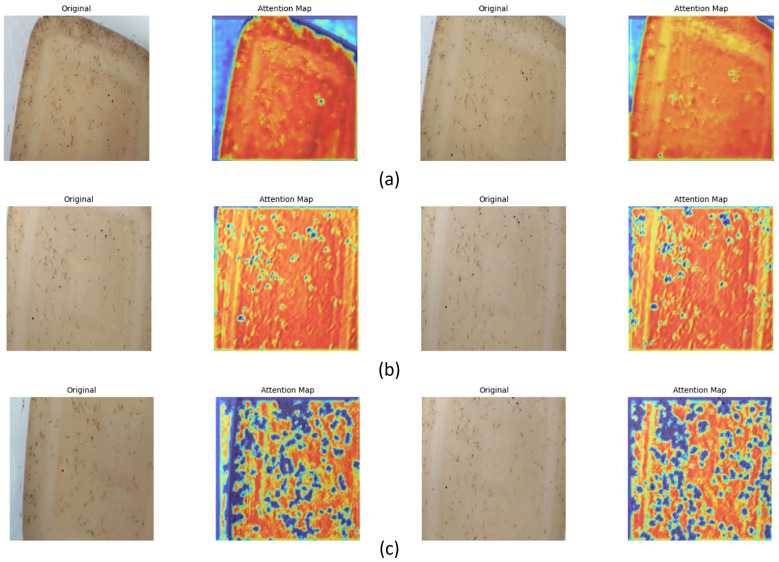
Grad-CAM visualization of PL5 under different augmentation strategies: **(a)** Baseline augmentation, **(b)** Moderate augmentation, and **(c)** Strong augmentation. Each row presents representative original images and their corresponding attention maps.

The Grad-CAM visualization of PL5 (Figure 13) clearly shows a progression in attention quality across the three employed augmentation conditions. The baseline concentrates activation on the background and edges, whereas moderate augmentation shows slight improvement in the attention toward PL. The proposed strong augmentation shows a highly clear concentration of attention on the larval body rather than the background or edges, and this confirms that the proposed model with strong augmentation suppresses the background dependency and focuses on feature learning on morphologically relevant features.

**Figure 14:**
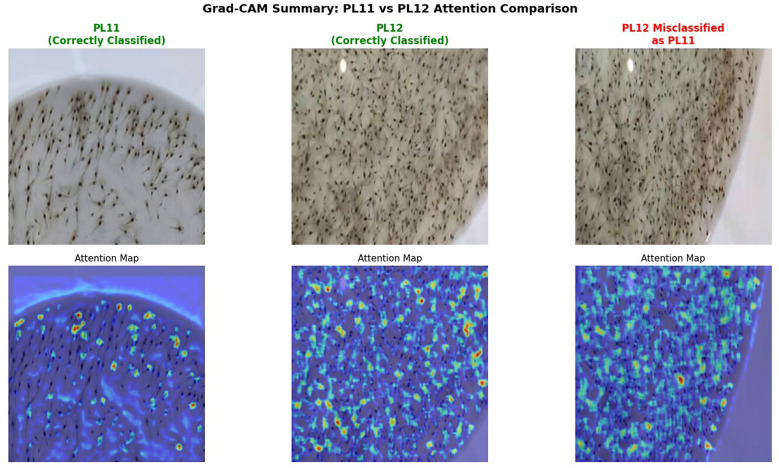
Grad-CAM attention maps for PL11 and PL12 classification, including a misclassified PL12 sample predicted as PL11.

To further address the PL11–PL12 classification boundary, Grad-CAM attention maps for correctly classified PL11, correctly classified PL12, and a misclassified PL12 sample (predicted as PL11) are presented in Figure 14. For correctly classified PL11, activation followed the spatial distribution of larval bodies across the bowl region, with localized hotspots on individual larvae. For correctly classified PL12, activation was strongly concentrated on individual larval bodies, reflecting confident stage-specific feature detection. For the misclassified PL12 sample, the activation is weak and diffuse, indicating insufficient discriminative feature detection, which is consistent with the morphological similarity between PL11 and PL12 discussed in Section 6.1.

### 6.5 HIDANet model ablation

#### 6.5.1 Model component analysis

In order to quantify the contribution of each architectural component, a systematic ablation study was performed over 100 training epochs with identical experimental conditions, and the results are shown in Table 13. The replacement of depthwise separable convolutions with standard convolutions increased the model parameters from 32,957 to 116,957 (~3.5 × increase), while the test accuracy was slightly reduced to 97.40%, thereby confirming the efficiency advantage of depthwise operations. The removal of the CBAM resulted in a test accuracy of 96.89%, thus revealing that joint channel–spatial refinement is crucial for discriminative feature learning. Furthermore, the removal of channel attention and spatial attention only reduced the performance to 94.29% and 93.94%, respectively, thereby proving that the two are complementary for structural localization. The elimination of ISOBlocks led to a test accuracy of 96.19%, thus pointing to their role in retaining fine-grained morphological representations. Similarly, the removal of the geometric channel progression caused the test accuracy to drop to 95.16%, thereby confirming its role in hierarchical feature abstraction. The full HIDANet architecture was able to reach a test accuracy of 98.28% with only 32,957 parameters, which was further increased to 98.44% under strong augmentation, thus showing that the integrated design is lightweight yet highly discriminative for morphology-driven classification.

**Table 13:** Evaluation of the impact of components in HIDANet on PL stage classification.

Model ablation (*e* = 100)	Params.	Train acc.	Valid acc.	Test acc.	Precision	Recall	F1-score
w/o Depthwise separable conv	116,957	97.16	99.15	97.40	0.97	0.98	0.98
w/o CBAM	31,319	85.92	93.77	96.89	0.93	0.95	0.94
w/o CA	31,613	88.03	96.42	94.29	0.94	0.96	0.95
w/o SA	32,663	93.46	97.27	93.94	0.94	0.95	0.94
w/o ISOBlocks	35,757	94.27	97.78	96.19	0.96	0.97	0.96
w/o channel progression	**28,845**	93.27	96.93	95.16	0.96	0.96	0.96
HIDANet (dropout = 0.3)	32,957	96.57	99.23	98.28	**0.99**	**0.99**	**0.99**
**HIDANet (dropout = 0.5) with strong augmentation**	32,957	**98.21**	**99.32**	**98.44**	**0.99**	**0.99**	**0.99**

Bold values indicate the best-performing result in each comparison.

#### 6.5.2 Architecture sensitivity analysis

To justify the design choices of the proposed HIDANet architecture, a sensitivity analysis was conducted by varying (i) the number of ISOBlocks, (ii) the number of branches per ISOBlock, and (iii) channel progression strategies. The results are summarized in Table 14.

**Table 14:** Impact of architectural configurations on HIDANet performance.

Configuration	Params. (M)	Test acc.	F1-score
ISOBlock depth (number of blocks)			
1 Block	**0.009**	88.93	0.88
2 Blocks	0.026	95.16	0.95
3 Blocks (proposed)	0.033	98.44	0.98
15.6-2.4,-1.3243pt4 Blocks	0.089	**99.65**	**0.99**
Number of branches per ISOBlock			
1 Branch	**0.026**	94.46	0.94
2 Branches (proposed)	0.033	**98.44**	**0.98**
15.6-2.4,-1.3243pt3 Branches	0.039	95.50	0.96
Channel progression (width scaling)			
Linear: 16 → 32 → 48 → 64	**0.015**	94.12	0.94
Progressive: 16 → 24 → 40 → 72	0.017	96.02	0.96
Geometric: 16 → 32 → 64 → 128 (Proposed)	0.033	98.44	0.98
Wide geometric: 16 → 32 → 80 → 224	0.066	**98.79**	**0.98**

Bold values indicate the best-performing result in each comparison.

For ISOBlock depth, increasing the number of blocks from one to three leads to a substantial improvement in performance (88.93% → 98.44%), indicating an enhanced feature representation capability. Although using four blocks achieves a slightly higher accuracy (99.65%), it significantly increases model complexity, reflecting diminishing returns. Therefore, three blocks provide an optimal trade-off between performance and efficiency.

For branch configuration, the use of two branches yields the best performance (98.44%), outperforming both single-branch and three-branch designs. This suggests that moderate multibranch feature extraction improves representational diversity without introducing redundancy.

For channel progression, the geometric scaling strategy (16 → 32 → 64 → 128) achieved a strong performance (98.44%) with efficient parameter utilization. Although a wider configuration (16 → 32 → 80 → 224) slightly improves the accuracy (98.79%), it comes at the cost of a notable increase in model complexity. Hence, the selected geometric progression offers a balanced design. Overall, the proposed configuration consisting of three ISOBlocks, two branches, and geometric channel scaling achieves an effective balance between classification accuracy and computational efficiency.

#### 6.5.3 Augmentation sensitivity analysis

An ablation study was conducted to evaluate the contribution of the individual augmentation components, as summarized in Table 15. The baseline model shows a substantial performance drop from 100.00% (RGB) to 66.26% (grayscale), corresponding to a large accuracy drop of 33.74%, indicating a strong reliance on color cues and significant background bias. Although moderate augmentation partially alleviates this issue, it still exhibits a notable drop (23.70%), suggesting that limited augmentation is insufficient to ensure robust generalization.

**Table 15:** Ablation study of HIDANet augmentation configurations showing RGB accuracy, grayscale robustness, and reduction in color dependency.

HIDANet augmentation configuration	Test acc. (%)	F1-score	Gray acc. (%)	Acc. drop (%)
Baseline augmentation	**100.00**	**100.00**	66.26	33.74
w/o RandomGrayscale	95.50	0.95	89.79	5.71
w/o ColorJitter	99.83	0.99	**99.83**	**–**
w/o RandomAutoContrast	98.96	0.98	98.44	0.52
Moderate augmentation	99.65	0.99	75.95	23.70
**Strong augmentation (proposed)**	98.44	0.99	96.89	1.55

Bold values indicate the best-performing result in each comparison.

In contrast, the ablated configurations significantly reduce this gap, with accuracy dropping to a maximum of 5.71%, demonstrating improved robustness to color variations. Notably, removing RandomGrayscale results in a relatively higher drop (5.71%) compared to the other components, indicating that grayscale augmentation plays a more critical role in enforcing color invariance. In comparison, removing ColorJitter or RandomAutoContrast has a minimal impact, suggesting that these augmentations provide complementary but less dominant contributions.

The proposed strong augmentation strategy achieves a balanced performance (98.44% RGB and 96.89% grayscale, with only a 1.55% drop), demonstrating that robustness arises from the combined effect of diverse augmentations. This design is motivated by real-world hatchery conditions, where PL-stage larvae exhibit significant visual variability owing to changes in illumination, background, and imaging environments, thereby necessitating color-invariant and morphology-driven feature learning.

### 6.6 Proposed HIDANet with existing model

#### 6.6.1 Model comparison and analysis of color dependency

Table 16 provides an overview of the classification results of the eight CNN models on the Vannamei shrimp post-larval stage classification dataset trained for 50 epochs. ResNet50 and EfficientNet-B0 scored 100.00% on the test, followed by SqueezeNet (98.79%) and MobileNetV2 (91.48%). The suggested HIDANet achieved a 97.23% test accuracy with 0.033M parameters, which is approximately 724 times less than that of ResNet50 (23.86M).

**Table 16:** Comparison of HIDANet with state-of-the-art models, epoch 50.

Model	Design paradigm	Valid acc.	Test acc.	Grayscale acc.	Accuracy drop	F1-score	Param (M)
MobileNetV3	Depthwise-separable CNN	98.38	97.58	72.97	24.94	0.98	3.120
MobileNetV2	NAS-optimized mobile CNN	98.55	91.48	77.14	14.34	0.86	2.431
EfficientNet-B0	Compound scaling	**100.00**	**100.00**	77.16	22.84	**1.00**	4.220
SqueezeNet	Ultra-light CNN	98.12	98.79	**95.85**	2.94	0.98	0.798
ShuffleNet-V2	Channel shuffle lightweight CNN	89.09	84.78	71.63	13.15	0.76	1.410
GhostNet	Ghost feature lightweight CNN	95.06	96.54	88.75	7.79	0.96	2.830
DenseNet121	Dense feature reuse	**100.00**	**100.00**	77.68	22.32	**1.00**	7.170
ResNet50	Residual learning	**100.00**	**100.00**	82.01	17.99	**1.00**	23.860
**HIDANet [proposed]**	**Lightweight CNN with strong augmentation**	98.89	97.23	94.46	**2.77**	0.98	**0.033**

Bold values indicate the best-performing result in each comparison.

In order to investigate the hypothesis that models rely on morphological but not chromatic information, test pictures were converted to luminance-equivalent three-channel grayscale inputs at the stage of evaluation only, and the resulting accuracy loss can be used as a proxy of color dependency. Although ResNet50, EfficientNet-B0, and DenseNet121 had 100% accuracy on color images, they decreased by 17.99%, 22.84%, and 22.32%, respectively. MobileNetV3-Small exhibited the largest decrease of 24.94%. GhostNet (Han et al., 2020) had a relatively weaker dependency (7.79%), and SqueezeNet had the lowest drop in baseline models (2.94%). HIDANet under baseline augmentation had the highest drop of any of the models considered (33.74%), whereas HIDANet with the proposed strong augmentation strategy dropped to 2.77%, maintaining 97.23% test accuracy and an RGB test-set F1-score of 0.98, highlighting that the proposed strong augmentation strategy effectively eliminates background color bias while preserving competitive classification performance.

Table 17 presents the HIDANet augmentation ablation retrained under 50-epoch conditions, which is consistent with the SOTA comparison in Table 16. Across all three augmentation variants, the grayscale accuracy improved progressively from 60.73% (baseline) to 94.46% (strong augmentation), even under 50-epoch training. This confirms that augmentation strength, rather than training duration, is the primary factor driving background bias elimination. These results are consistent with the 100-epoch ablation reported in Table 11, which additionally includes Cohen's kappa and MCC metrics for a comprehensive evaluation.

**Table 17:** HIDANet augmentation ablation under 50-epoch training conditions.

Variant	Valid acc. (%)	Test acc. (%)	Grayscale acc. (%)	Acc. drop (%)	F1
HIDANet — Baseline Aug	99.40	98.62	60.73	37.89	**0.99**
HIDANet—Moderate Aug	**99.91**	**99.13**	63.84	35.29	**0.99**
**HIDANet—Strong Aug [proposed]**	98.89	97.23	**94.46**	**2.77**	0.98

Bold values indicate the best-performing result in each comparison.

#### 6.6.2 Statistical comparison with state-of-the-art models

Table 18 summarizes the statistical comparison between HIDANet and state-of-the-art CNN models using test accuracy, 95% bootstrap confidence intervals (Efron and Tibshirani, 1994), and McNemar's test (Oyeka, 2012). HIDANet achieved 97.23% accuracy with a 95% CI of [95.85%, 98.44%], confirming stable and reliable performance across the test set (*n* = 578).

**Table 18:** Statistical comparison of HIDANet with state-of-the-art models under RGB and grayscale conditions (*n* = 578).

Model	Test acc. (%)	Gray acc. (%)	95% CI (bootstrap)	McNemar (*p*)	Sig.	Interpretation
ShuffleNet-V2	84.78	71.63	[81.76%, 87.80%]	< 0.001	^***^	Significantly worse
MobileNetV2	91.48	77.14	[89.16%, 93.77%]	< 0.001	^***^	Significantly worse
GhostNet	96.54	88.75	[94.98%, 97.92%]	0.6171	ns	Comparable
MobileNetV3	97.58	72.97	[96.37%, 98.62%]	0.7893	ns	Comparable
SqueezeNet	98.79	95.85	[97.75%, 99.65%]	0.0339	^*^	Slightly better
ResNet50	**100.00**	82.01	**[99.34%, 100.00%]**	0.1573	ns	Comparable
DenseNet121	**100.00**	77.68	**[99.34%, 100.00%]**	< 0.001	^***^	Higher RGB; color dependent
EfficientNet-B0	**100.00**	77.16	**[99.34%, 100.00%]**	< 0.001	^***^	Higher RGB; color dependent
HIDANet (Proposed)	97.23	94.46	[95.85%, 98.44%]	–	–	Reference

***p < 0.001, *p < 0.05, ns, not significant. McNemars test evaluates paired prediction disagreement; bootstrap confidence intervals provide complementary uncertainty estimation. Bold values indicate the best-performing result in each comparison.

The proposed HIDANet significantly outperforms ShuffleNet-V2 and MobileNetV2 (*p* < 0.001), while achieving statistically equivalent test accuracy to MobileNetV3, GhostNet, and ResNet50 (*p*≥0.05) at substantially fewer parameters. SqueezeNet demonstrated a marginal statistical advantage in test accuracy (*p* = 0.0339); however, HIDANet achieved comparable grayscale robustness at 24 × fewer parameters, confirming its practical deployment superiority. DenseNet121 and EfficientNet-B0 achieve perfect RGB accuracy (100%, *p* < 0.001); however, their grayscale accuracy drops to 22.32% and 22.84%, respectively, confirming strong color dependency, in contrast to HIDANet, which maintains 94.46% grayscale accuracy with an accuracy drop of only 2.77%—the lowest among all models evaluated. This confirms that the proposed strong augmentation strategy effectively eliminates background color bias while maintaining competitive classification performance.

### 6.7 Computational efficiency analysis of HIDANet

To assess the computational efficiency of the proposed HIDANet architecture, a comprehensive complexity analysis was performed. Table 19 summarizes the model parameters, FLOPs, memory footprints, and inference latencies. Comparisons with popular lightweight CNNs, such as MobileNetV3, ShuffleNetV2, MobileNetV2, SqueezeNet, GhostNet, EfficientNet, DenseNet121, and ResNet50, were performed with the same FP32 inference settings using the 224 × 224 input resolution and a batch size of 1. As shown in Table 20, the proposed HIDANet demonstrates superior computational efficiency compared with conventional lightweight CNNs across multiple efficiency metrics, such as P_*max*_, FLOPs_*max*_, and F_*max*_. The inference latency is measured with identical hardware and batch sizes for a fair comparison.

**Table 19:** Performance and computational efficiency metrics.

Metric	Method of calculation	Remarks
Model parameters	$P_{\text{total}}=\overset{L}{\underset{l=1}{\sum }}P_{l}$	Sum of all trainable parameters across layers.
Fully connected layer parameters	*P*_FC_ = (*n*_in_×*n*_out_)+*n*_out_	Parameters for a fully connected layer with *n*_in_ inputs and *n*_out_ outputs.
FLOPs (standard convolution)	${\text{FLOPs}}_{\text{conv}}=2\times C_{\text{in}}\times C_{\text{out}}\times k^{2}\times H_{\text{out}}\times W_{\text{out}}$	Floating-point operations for convolution.
FLOPs (depthwise separable convolution)	${\text{FLOPs}}_{\text{DW+PW}}=\left(2\times C_{\text{in}}\times k^{2}\times H_{\text{out}}\times W_{\text{out}}\right)+\left(2\times C_{\text{in}}\times C_{\text{out}}\times H_{\text{out}}\times W_{\text{out}}\right)$	Total FLOPs for depthwise and pointwise convolutions.
FLOPs (fully connected layer)	FLOPs_FC_ = *B*×(2 × *n*_in_×*n*_out_−*n*_out_)	Computational cost of dense layers; *B* is the batch size.
Total GFLOPs	$\text{GFLOPs}=\frac{\underset{l}{\sum }{\text{FLOPs}}_{l}}{10^{9}}$	Total floating-point operations across all layers, expressed in giga-FLOPs.
Model size	$S=\frac{P_{\text{total}}\times 32}{8\times 10^{6}}\;\text{MB}$	Memory footprint of the model (assuming 32-bit floating-point representation).
Inference latency	$T_{\text{latency}}=\frac{1}{N_{\text{runs}}}\overset{N_{\text{runs}}}{\underset{r=1}{\sum }}t_{r}\;\text{(ms)}$	Average inference time per image over *N*_runs_ test runs.
Frames per second (FPS)	$\text{FPS}=\frac{1,000}{T_{\text{latency}}}$	Throughput measured in frames per second (latency in ms).
Parameter efficiency score	$S_{P}=\left(1-\frac{P_{\text{total}}}{P_{\max}}\right)\times 100$	Normalized parameter efficiency relative to the largest model.
Computational efficiency score	$S_{C}=\left(1-\frac{{\text{GFLOPs}}_{i}}{{\text{GFLOPs}}_{\max}}\right)\times 100$	Normalized FLOPs efficiency across all evaluated models.
Speed efficiency score	$S_{V}=\left(\frac{{\text{FPS}}_{i}}{{\text{FPS}}_{\max}}\right)\times 100$	Normalized inference speed.
Overall efficiency score	$S_{\text{overall}}=\frac{S_{P}+S_{C}+S_{V}}{3}$	Unified efficiency metric combining model size, computation, and speed; ranges from 0 (least efficient) to 100 (most efficient).

**Table 20:** Evaluation of computational efficiency of proposed HIDANet model.

Model	Params. (M)	Params. (MB)	GFLOPs	Latency (ms)	FPS	Overall efficiency score
MobileNetV3-Small	1.53	5.82	0.11	5.10	196.18	79.2
ShuffleNetV2	1.26	4.81	0.29	6.35	157.41	75.7
MobileNetV2	2.23	8.52	0.60	5.08	196.97	76.5
SqueezeNet	0.73	2.77	0.53	**2.05**	**486.63**	**97.4**
GhostNet	2.83	10.69	0.16	11.47	87.22	67.98
EfficientNet-B0	4.02	15.32	0.77	7.93	126.06	69.6
DenseNet121	6.96	26.55	5.67	15.48	64.59	43.3
ResNet50	23.52	89.73	8.17	5.95	168.13	29.3
HIDANet [Proposed/GPU]	**0.03**	**0.12**	0.34	2.74	364.87	90.9
HIDANet [Proposed/CPU]	**0.03**	**0.12**	0.34	25.17	39.73	67.96

Bold values indicate the best-performing result in each comparison.

With only 0.033 M parameters (0.12 MB) and 0.34 GFLOPs, the HIDANet achieves a latency of 2.74 ms with a high frame rate of 364.87 FPS, yielding an overall efficiency score of 90.9. In contrast, well-established compact models such as MobileNetV3 (79.2) and ShuffleNetV2 (75.7) exhibit higher parameter counts and computational demands, and although SqueezeNet achieves the highest efficiency score of 97.4, it requires 0.798M parameters, approximately 24 times more than the proposed model, and exhibits greater background color dependency (grayscale accuracy drop: 2.94% vs. 2.77%, Table 16). However, the proposed model balances accuracy, robustness, and efficiency with substantially fewer parameters.

The larger models included DenseNet121 (43.3) and ResNet50 (29.3), all with significantly higher latency and resource usage. The proposed HIDANet with its lightweight design achieves effective performance through its built-in multibranch structure, which includes parallel branching, CBAM attention modules, and depthwise separable convolutions.

Based on the experiment, the top five lightweight architectures, namely, MobileNetV3, ShuffleNetV2, the proposed HIDANet, SqueezeNet, and MobileNetV2, were identified, confirming that the proposed HIDANet with strong augmentation is computationally efficient. Furthermore, to assess the deployment feasibility on resource-constrained hardware, an additional CPU inference benchmark for the proposed HIDANet model was conducted in a cloud-based x86-64 virtualized environment. Here, the proposed HIDANet achieves a mean inference latency of 25.17 ms and 39.73 FPS on the CPU, exceeding the standard real-time video processing threshold of 25 30 FPS, with a model footprint of only 0.12 MB and 0.033M parameters. The existing compact models range between 0.73M and 4.21M parameters, whereas the proposed model is approximately 77 × , 74 × , and 43 × smaller than MobileNetV2, MobileNetV3, and ShuffleNet-V2, respectively. The combination of 25.17 ms CPU latency, 39.73 FPS throughput, and 0.12 MB model footprint confirms that the proposed HIDANet satisfies the computational constraints required for real-time deployment in resource-constrained aquaculture monitoring applications.

### 6.8 Integrated larvae prediction and morphometric estimation

#### 6.8.1 Prediction and counting of PL developmental stages

The proposed analytical framework integrates developmental stage prediction, automated larval detection, and morphometric analysis from digital PL images. The trained classifier can accurately predict the development stage with a high level of confidence (e.g., PL9 with 98.73% confidence), thus demonstrating the feasibility of the proposed model for hatchery use.

For density estimation, the image processing pipeline consisted of contrast enhancement using CLAHE and adaptive thresholding for larval segmentation from the background. The initial contour extraction phase resulted in a large number of candidate regions (e.g., 2,097 contours). By applying skeleton-based filtering and geometric constraints, invalid regions, including debris and noise, were removed, and 349 valid larval regions were extracted from the sample image.

**Figure 15:**
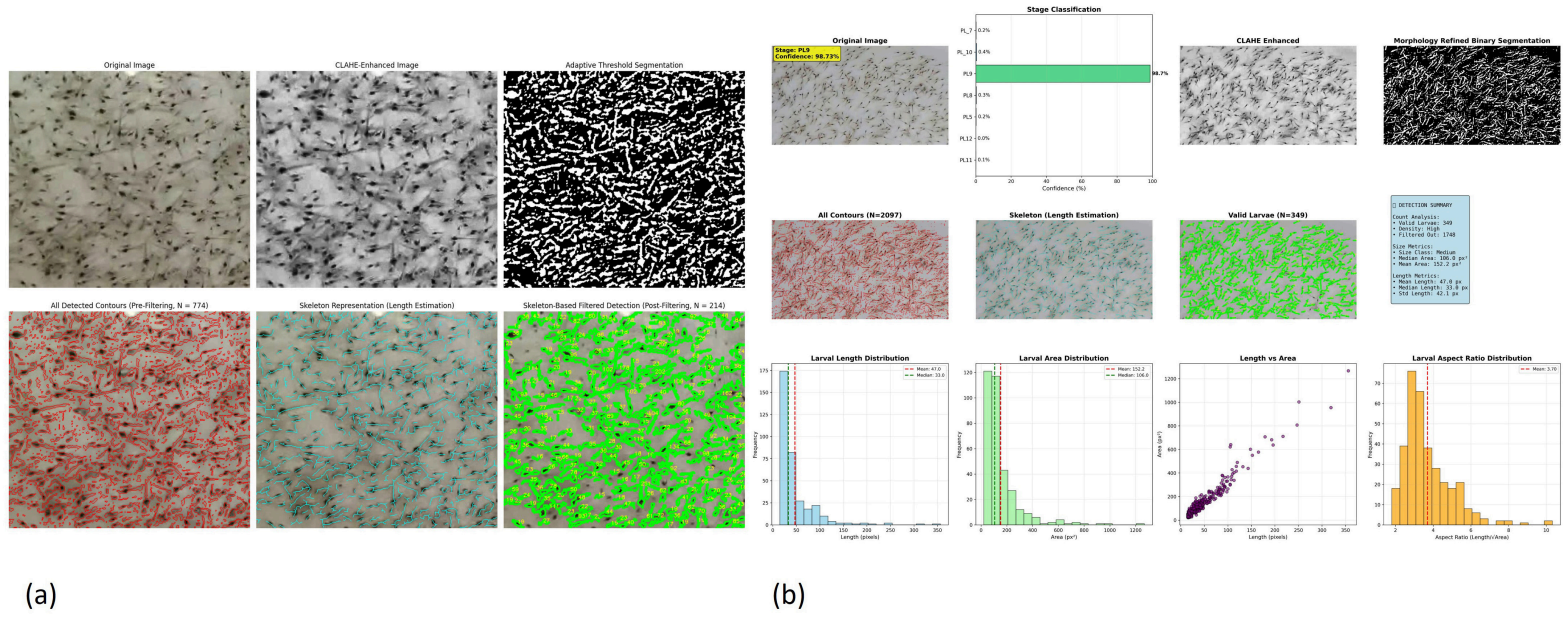
Integrated pipeline for shrimp post-larval stage classification and morphometric analysis: **(a)** skeleton-based larval detection and filtering workflow, and **(b)** PL stage classification with confidence scores and morphometric distributions.

Morphometric estimation, as shown in Figures 15a, b, was also performed by extracting skeleton-based body length using the Zhang-Suen thinning algorithm (Zhang and Suen, 1984), and contour area from binary-segmented larval instances. The detected larvae had a mean skeleton length of approximately 47 px, with a median of 33 px, thus showing a certain level of variation in larval posture and orientation. The contour area also followed a similar trend, with a mean area of approximately 152 px^2^ and a median of 106 px^2^, thus showing a moderate level of variation in body size between the observed populations. These measurements were reported in pixel units, as spatial calibration data were not recorded during image acquisition. Therefore, physical unit conversion is identified as a limitation of the current morphometric pipeline and is discussed in Section 7.2.

#### 6.8.2 Quantitative evaluation of larvae counting accuracy

To validate the morphometric counting pipeline, ground-truth (GT) counts were obtained by manually annotating 120 images using the Roboflow annotation platform, yielding 29,731 annotated larvae across the evaluation set. The automated pipeline was applied to the same images, and predictions were compared against GT using four metrics: mean absolute error (MAE, larvae per image), root mean squared error (RMSE), mean absolute percentage error (MAPE), and Pearson correlation coefficient (*r*). The quantitative results are summarized in Table 21.

**Table 21:** Quantitative evaluation of the automated larvae counting pipeline against Roboflow-annotated ground truth (*n* = 120 images; 29,731 GT larvae).

Images	Total GT	Total Pred	MAE (larvae/img)	RMSE	MAPE (%)	Pearson *r*
120	29,731	32,631	40.65	50.11	21.87	0.9007

The pipeline predicted 32,631 larvae compared to a manual GT of 29,731, corresponding to a difference of 9.7%. However, given the high larval density, frequent overlap among individuals, and inherent optical blur in hatchery imaging conditions (Zhou et al., 2024; Duan et al., 2024; Bukas et al., 2024), manual annotation is prone to systematic undercounting. Annotators may miss larvae in occluded or low-contrast regions, which is a well-recognized limitation in dense biological counting tasks (Peng et al., 2024; Qian et al., 2023; Wang et al., 2024).

Consequently, the observed discrepancy likely reflects under-annotation in the ground truth rather than true over-prediction using an automated pipeline (Bukas et al., 2024; Zhou et al., 2024). The strong Pearson correlation (*r* = 0.9007) further confirms that the pipeline reliably tracks relative larval abundance across images, which is an operationally relevant requirement for hatchery quality control applications (Racotta et al., 2004; Dang et al., 2025; Correia et al., 2025). These findings show that the proposed system may offer quantitative population metrics and precise classification of developmental stages with automated evaluation of larval density and morphological traits in hatchery settings.

### 6.9 Deployment robustness

To address practical deployment concerns, a simulated robustness evaluation was conducted on the held-out test set without retraining. Six degradation conditions representative of real-world hatchery imaging variability were applied as inference-time transformations, including low lighting, low resolution, motion blur, sensor noise, water turbidity, and combined degradation (simultaneous lighting variation, blur, and noise). The baseline accuracy of the proposed HIDANet (98.44%) was used as a reference, and the results are summarized in Table 22.

**Table 22:** Simulated deployment robustness evaluation of HIDANet under representative hatchery imaging degradations.

Condition	Accuracy (%)	Drop from proposed (%)
HIDANet (proposed)	98.44	0.00
Low light	97.06	−1.38
Motion blur	99.13	+0.69
Sensor noise	98.10	−0.34
Water turbidity	96.71	−1.73
Combined degradation	90.66	−7.78
Low resolution	73.53	−24.91

The model demonstrates strong robustness across four out of six conditions, maintaining an accuracy above 96% under low light, motion blur, sensor noise, and water turbidity, confirming stability under common hatchery imaging challenges. Under combined degradation, the accuracy remains at 90.66% (drop: −7.78%), indicating reasonable resilience to multiple simultaneous distortions. However, the reduced resolution condition (112 × 112) resulted in a notable performance drop to 73.53% (drop: −24.91%), suggesting that image resolution is a critical factor influencing classification reliability.

Overall, these results confirm the robustness of the proposed pipeline under simulated real-world imaging variability without retraining or fine-tuning. The model maintained high performance under most conditions and demonstrated strong generalization capability, indicating its suitability for deployment across diverse hatchery environments beyond the single facility used for data collection.

## 7 Discussion and limitation

### 7.1 Discussion

The results highlight the limitations of using classification accuracy as the sole criterion for model assessment in biological imaging problems. Although they achieved perfect RGB accuracy, the strongest baseline HIDANet showed the largest grayscale accuracy drop of 33.74% and the lowest color–gray cosine similarity of 0.61 among all configurations, thus proving that the model learned from background color information rather than from post-larval morphology. This observation was consistent across all pre-trained models, such as ResNet50, EfficientNet-B0, and DenseNet121, which all achieved perfect RGB accuracy but experienced grayscale accuracy drops of over 17%, thus proving that neither model complexity nor ImageNet pre-training can prevent models from learning shortcuts in domain-specific biological datasets.

The proposed method of strong data augmentation successfully countered this bias without adding new parameters or changing the architecture. The grayscale accuracy drop decreased from 33.74% to 1.55%, the color–gray cosine similarity increased to 0.96, and ARI and NMI increased to 0.945 and 0.948, respectively, which is the highest among all augmentation settings, thus proving that the model learned color-invariant, morphology-driven features. These gains were achieved while retaining 98.44% test accuracy and an F1-score of 0.984 and were confirmed to be stable across three-fold cross-validation with a mean validation accuracy of 97.77%±0.43.

The ablation study also confirms that the removal of CBAM has the most significant effect on this performance, with the largest impact on accuracy (reduced to 96.89%) and F1-score (reduced to 0.94) the most. The other parts, ISOBlocks, channel progression, and depthwise separable convolution, all have an incremental effect, thus justifying the overall architecture design.

In the context of implementation, the proposed HIDANet model is computationally efficient with 364.87 FPS and 2.74 ms latency and only 0.033M parameters, which is not possible with larger architectures despite their similar accuracy. Overall, the study demonstrates that robust PL stage classification requires explicit bias control through augmentation and that robustness metrics such as grayscale accuracy drop, MCC, and feature-space similarity should complement conventional accuracy evaluation in biological imaging tasks.

### 7.2 Limitations and future direction

All experiments in this study were conducted using images from a single hatchery environment. Although hatchery setups are generally standardized, minor variations in imaging conditions, such as lighting, camera characteristics, and water properties, may still influence model performance. To partially address this, a strong augmentation strategy (including color jitter, grayscale conversion, and auto-contrast) was employed to improve the robustness to such variations. In addition, robustness was evaluated through simulation-based image degradation on a held-out test set. However, explicit cross-domain validation of the independently collected datasets was not performed in the current study. Evaluating the model on data from different hatchery environments remains an important direction for future work to further confirm the generalization capability.

Additionally, the proposed work classifies seven post-larval developmental stages (PL5 and PL7–PL12) and explicitly excludes PL6, as specimens at this stage were unavailable during the data collection period owing to staggered stocking schedules at the source facility. Despite specific inquiries on the day of visits and requests for access on subsequent days, PL6 specimens were not present during the entire data collection period. Hatchery personnel further confirmed that PL5 and PL6 are not consistently distinguished during routine staging and labeling operations at the facility. Therefore, the system is not intended for deployment in hatcheries that require explicit PL6 identification without retraining on PL6-inclusive data. PL6 data collection across multiple hatchery facilities was identified as a primary direction for future work to achieve complete developmental stage coverage and full commercial deployability.

CPU inference benchmarks conducted on a cloud-based x86_64 virtualized environment (Google Colab Pro; typically Intel Xeon/AMD EPYC-class CPUs) indicate that HIDANet achieves 39.73 FPS with 25.17 ms latency. However, as these results were obtained on virtualized server-grade hardware rather than dedicated embedded devices, benchmarking on representative embedded hardware under hatchery conditions remains an important direction for future work.

The current morphometric pipeline successfully extracted skeleton-based body length and contour area measurements from segmented larval instances, demonstrating the feasibility of automated morphometric estimation from hatchery images. As spatial calibration data were not recorded during image acquisition at the source hatchery, measurements are currently reported in pixel units and cannot be directly compared to the absolute body length references used in commercial PL staging protocols. Establishing a pixel-to-millimeter conversion through imaging system calibration is identified as a natural and straightforward extension of the current pipeline, which will enable operationally meaningful morphometric reporting in future work.

## 8 Conclusion

The proposed study involved HIDANet, a small-sized deep learning architecture that could be used to classify the stages of post-larval shrimp *Penaeus vannamei* development in the natural setting of imaging in the hatchery. It is based on the integration of effective convolutional processes and hierarchical feature release to generate both high discriminative ability and very low computational complexity. Numerous experiments were conducted to test the performance in terms of classification and computational efficiency.

A comparative study with some commonly used CNN models demonstrated that despite their ability to approach perfect accuracy with large networks, such as ResNet, DenseNet, and EfficientNet, the latter need significantly more computation units, and HIDANet was competitive in terms of accuracy with fewer parameters of 0.033M and was much more viable for deployment in real aquaculture settings.

Background bias analysis with a grayscale test showed that traditional training systems are prone to using color information related to hatchery conditions. The proposed approach enhanced robustness through the introduction of a stronger augmentation strategy, which also promoted the use of morphological features instead of background artifacts by the model. This observation demonstrates the relevance of bias-conscious training methods in the classification of biological images. In addition to stage prediction, a pipeline of integrated analysis was created to detect, count, and estimate the morphometrics of larvae. The framework was able to isolate valid larval samples and measure both structural traits (skeleton length and distributions of contour areas) and is a useful measure of both larval population density and developmental heterogeneity.

The possibility of computer analysis also provides evidence that HIDANet has a good balance between speed, model size, and accuracy, allowing real-time inferencing and preserving a high classification rate. Altogether, the proposed framework outlines a convenient and expandable solution to automated shrimp hatchery monitoring and proves the possibility of lightweight AI systems for aquaculture applications. The upcoming development will involve the expansion of the dataset to various hatcheries, better domain generalization, and automated aquaculture management systems through real-time deployment.

## Data Availability

The raw data supporting the conclusions of this article will be made available by the authors, without undue reservation.
